# Correction to: Transcriptional responses of *Escherichia coli* during recovery from inorganic or organic mercury exposure

**DOI:** 10.1186/s12864-018-4631-z

**Published:** 2018-04-18

**Authors:** Stephen P. LaVoie, Anne O. Summers

**Affiliations:** Department of Microbiology, University of Georgia, Athens, GA 30602 USA

**Correction**

After publication of the original article [1] the authors noted that the key displayed in Figure 1a was incorrect, as the PMA and HgCl2 conditions had been switched.

The correct information follows below:

**MG1655 unexposed condition**: blue line

**MG1655 + 3μM PMA**: green line

**MG1655 + 3μM HgCl2**: red line.

The revised figure is included with this Correction (Fig 1).

**Corrected Figures**:

**Revised Fig.** **1**:

**Fig. 1:**
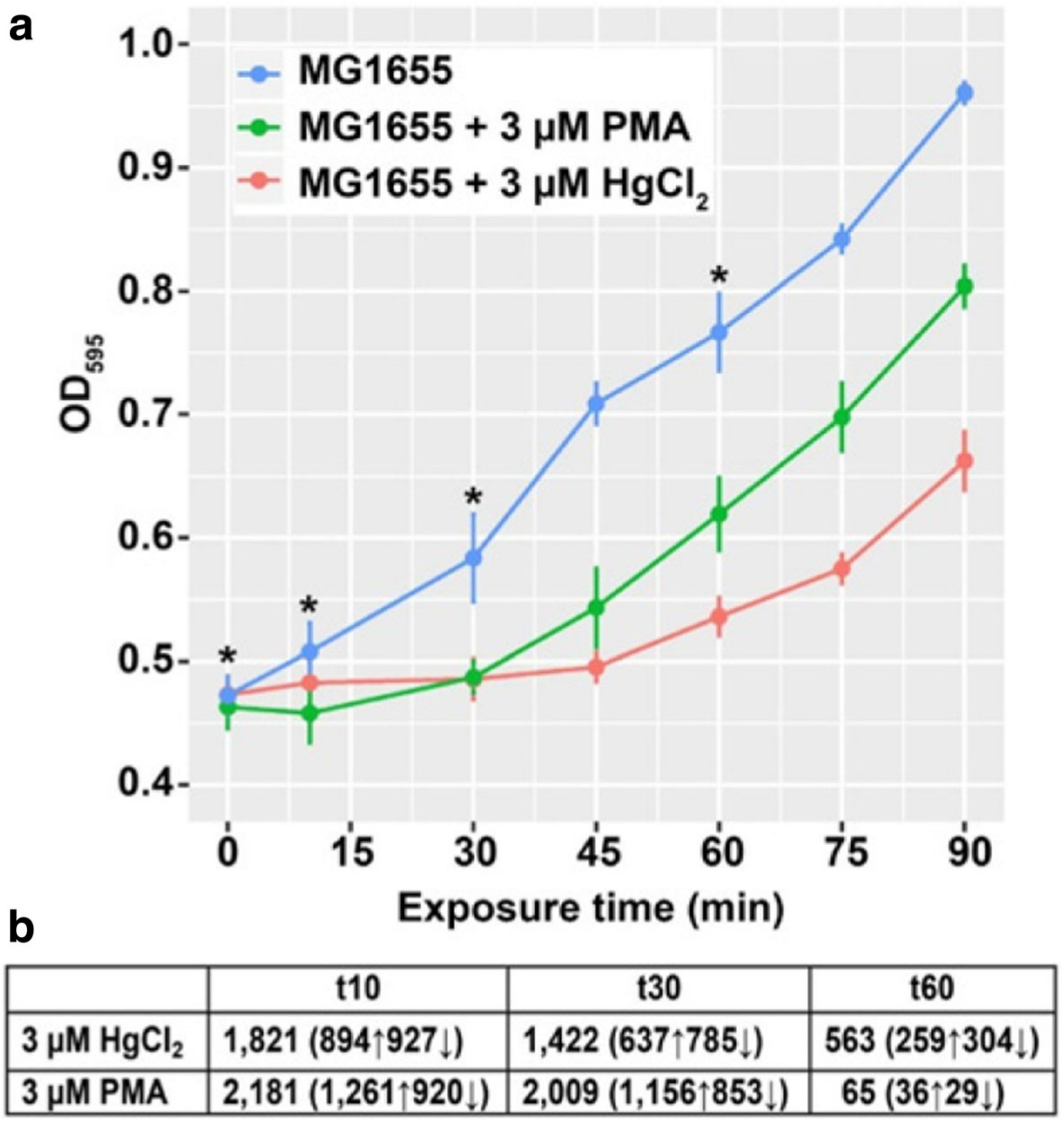
Effects of sub-acute mercury exposure on growth of MG1655. **a** *E. coli* K12 MG1655 grown in MOPS minimal medium, unexposed (blue) or exposed to 3 μM HgCl_2_ (red) or 3 μM PMA (green) during mid-log phase. Asterisks indicate sampling times for RNA-seq. Error bars are standard error (SEM) of 3 biological replicates for each culture condition. See Additional file 1: Figure S1 for full growth curve. **b** Significantly differentially expressed genes (DEG) counts (up-regulated or down-regulated) for HgCl_2_ and PMA relative to unexposed control culture at each time point
