# Supplementary material for: Transcriptional responses of Escherichia coli during recovery from inorganic or organic mercury exposure
Source: BMC Genomics. 2018 Jan 16;19:52. doi: 10.1186/s12864-017-4413-z (PMC5769350; doi:10.1186/s12864-017-4413-z)
Supplement: Supplementary file 1 — Contains supporting methods, Tables S1 - S3, Table legends for Tables S4 - S17, and Figures S1 - S29. (PDF 13721 kb) [file 12864_2017_4413_MOESM1_ESM.pdf]

Supporting Material:

Transcriptional responses of *Escherichia coli* during recovery from inorganic or organic mercury exposure

Stephen P. LaVoie* and Anne O. Summers*

Department of Microbiology, University of Georgia, Athens, Georgia 30602 USA

Supporting Methods:

RNA*snap*™ method [1].

Each thawed cell pellet was suspended in 100µl of RNA*snap* extraction solution (95% formamide (RNA grade), 18mM EDTA (pH 8), 0.025% SDS, 1% βME), then vortex vigorously. The cells were then lysed by heating at 95°C in a heat block for 7 min. Cell debris was pelleted by centrifuging the warm sample at 16,000 *g* for 5 min at room temperature. 90 µl of supernatant was then transferred to a fresh tube, without disturbing clear gelatinous pellet. RNA was quantified with Nanodrop™ using A_{260} and RNA*snap* solution for blank. The RNA was then further isolated using phenol/chloroform extraction, by adding 340 µl of RNase free H_2O and 430 µl of acidic phenol/chloroform (5:1 solution, pH 4.5, Ambion) and vortex vigorously for 30 sec. Each sample was centrifuged at 21,000 x *g* for 15 min at 4°C, then 350 µl of the aqueous phase was transferred to a fresh tube. Then to precipitate RNA, 40 µl of sodium acetate (3 M, pH 5.5) and 1 ml of 100% ethanol (~2.5x volume) was added and mixed by inverting the tube several times. Samples were precipitated for 1 hr at -70°C and moved to -20°C to continue precipitation overnight. The precipitated samples were centrifuged at 21,000 *g* for 15 min at 4°C to pellet RNA, then the supernatant was carefully removed by aspiration, the pellet was washed with 300 µl of 70% ethanol and centrifugation was repeated. The supernatant was carefully removed by aspiration, the RNA pellet was air dried completely, and allowed at least 30 min to hydrate in 50 µl of RNase free H_2O before quantifying using Nanodrop™ to determine recovery. RNA samples were then stored at -70°C until DNase treatment. RNA quality was assessed after DNase treatment by running 500 ng of RNA on 1% agarose – 0.5 X TBE gel with 0.5 µg/ml

ethidium bromide, run at 50V for 2 hr. RNA samples were denatured prior to loading by suspension in Gel Loading Buffer II (95% formamide, 18mM EDTA and 0.025% each of SDS, xylene cyanol and bromophenol blue, Ambion) and heated for 5 min. at 95°C.

Quantification of cell mercury content

A single cell pellet for each condition and time point was collected and cryoarchived as described in the Cell Cultivation section above. Frozen sample tubes were shipped overnight on dry ice to Rutgers University for analysis. Cell pellets were digested following EPA method 245.6 with the exception that potassium persulfate was not used during the oxidation process. Total Hg was quantified with a Hydra II cold vapor atomic absorption (CVAA) instrument (Teledyne-Leeman Labs) using an external standard curve for each run. Data were collected from three biological replicates prepared by the same method, but not used for RNA-Seq analysis. The average background mercury from flask contamination in unexposed samples across all time points (10.10 ng/pellet +/- 2.55) was subtracted from mercury exposed cell pellet values. Total input mercury (200.59 g/mol) is based on 3 μM Hg added to culture equals 3×10^{-9} moles/ml or 602 ppb, and was used to determine the percentage of input mercury bound by cells at each time point in $HgCl_2$ and PMA exposed cells.

Supporting Tables:

Table S1: Sequencing index tag for each sample library.

Sample ID	index	sequence
1A (MG_t0)	701-501	ATTACTCG-TATAGCCT
2A (MG_t10)	701-502	ATTACTCG-ATAGAGGC
3A (MG_t30)	701-503	ATTACTCG-CCTATCCT
4A (MG_t60)	701-504	ATTACTCG-GGCTCTGA
5A (MG+Hg_t10)	701-505	ATTACTCG-AGGCGAAG
6A (MG+Hg_t30)	701-506	ATTACTCG-TAATCTTA
7A (MG+Hg_t60)	701-507	ATTACTCG-CAGGACGT
8A (MG+PMA_t10)	701-508	ATTACTCG-GTACTGAC
9A (MG+PMA_t30)	702-501	TCCGGAGA-TATAGCCT
10A (MG+PMA_t60)	702-502	TCCGGAGA-ATAGAGGC
1B (MG_t0)	702-503	TCCGGAGA-CCTATCCT
2B (MG_t10)	702-504	TCCGGAGA-GGCTCTGA
3B (MG_t30)	702-505	TCCGGAGA-AGGCGAAG
4B (MG_t60)	702-506	TCCGGAGA-TAATCTTA
5B (MG+Hg_t10)	702-507	TCCGGAGA-CAGGACGT
6B (MG+Hg_t30)	702-508	TCCGGAGA-GTACTGAC
7B (MG+Hg_t60)	703-501	CGCTCATT-TATAGCCT
8B (MG+PMA_t10)	703-502	CGCTCATT-ATAGAGGC
9B (MG+PMA_t30)	703-503	CGCTCATT-CCTATCCT
10B (MG+PMA_t60)	703-504	CGCTCATT-GGCTCTGA
1C (MG_t0)	703-505	CGCTCATT-AGGCGAAG
2C (MG_t10)	703-506	CGCTCATT-TAATCTTA
3C (MG_t30)	703-507	CGCTCATT-CAGGACGT
4C (MG_t60)	703-508	CGCTCATT-GTACTGAC
5C (MG+Hg_t10)	704-501	GAGATTCC-TATAGCCT
6C (MG+Hg_t30)	704-502	GAGATTCC-ATAGAGGC
7C (MG+Hg_t60)	704-503	GAGATTCC-CCTATCCT
8C (MG+PMA_t10)	704-504	GAGATTCC-GGCTCTGA
9C (MG+PMA_t30)	704-505	GAGATTCC-AGGCGAAG
10C (MG+PMA_t60)	704-506	GAGATTCC-TAATCTTA

Table S2: Cell mercury content.

Condition (replicates)	Hg Content (%CV) (ng/pellet)	Percent of Input Hg (602 ng)
MG+Hg_t10 (5)	296.70 (62%)	49%
MG+Hg_t30 (6)	144.42 (34%)	24%
MG+Hg_t60 (6)	279.90 (49%)	47%
MG+PMA_t10 (5)	277.90 (14%)	46%
MG+PMA_t30 (6)	105.42 (73%)	18%
MG+PMA_t60 (6)	63.30 (36%)	11%

Table S3: Average total mapped reads by feature type from 3 biological replicates for each condition.

	MG_t0	MG_t10	MG_t30	MG_t60	Hg_t10	Hg_t30	Hg_t60	PMA_t10	PMA_t30	PMA_t60
**sum of counts for all feature types (4,495 total)**	6304455	6216680	6471741	6708912	7060100	6407018	7594243	7617725	5306298	6674420
**sum of CDS features (4,140 total)**	5637398 (89%)	5600499 (90%)	5756229 (89%)	5824959 (87%)	6264405 (89%)	5362239 (84%)	6377199 (84%)	6535950 (86%)	4446307 (84%)	5940351 (89%)
**sum of ncRNA features (63 total)**	226511 (3.6%)	219188 (3.5%)	259520 (4.0%)	326701 (4.9%)	350434 (5.0%)	436892 (6.8%)	484101 (6.4%)	420290 (5.5%)	313073 (5.9%)	248544 (3.7%)
**sum of pseudogene features (178 total)**	34727 (0.6%)	32490 (0.5%)	38669 (0.6%)	39796 (0.6%)	16693 (0.2%)	15572 (0.2%)	24078 (0.3%)	54826 (0.7%)	41601 (0.8%)	36088 (0.5%)
**sum of rRNA features (22 total)**	24956 (0.40%)	14601 (0.23%)	17620 (0.27%)	18935 (0.28%)	1510 (0.02%)	25925 (0.40%)	364 (0.01%)	4010 (0.05%)	29871 (0.56%)	23752 (0.36%)
**sum of tRNA features (86 total)**	42271 (0.67%)	21539 (0.35%)	17451 (0.27%)	17998 (0.27%)	13814 (0.20%)	16077 (0.25%)	39166 (0.52%)	17836 (0.23%)	13134 (0.25%)	20339 (0.30%)
**sum of tmRNA features (2 total)**	338581 (5%)	328355 (5%)	382239 (6%)	480511 (7%)	413212 (6%)	550291 (9%)	669320 (9%)	584792 (8%)	462282 (9%)	405337 (6%)
**sum of tRNA-pseudogene features (3 total)**	11 (<0.1%)	7 (<0.1%)	13 (<0.1%)	11 (<0.1%)	31 (<0.1%)	22 (<0.1%)	15 (<0.1%)	22 (<0.1%)	30 (<0.1%)	9 (<0.1%)

Average total reads from 3 biological replicate libraries for each condition. Sums and average for each feature type are based on the annotation file Escherichia_coli_str_k_12_substr_mg1655.GCA_000005845.2.24.gtf. Percentages were determined by dividing the average total reads for each feature type by the average total reads for all features in each library.

Supporting Table Footnotes:

Table S4: Raw counts. Read counts for each gene were determined by counting mapped paired-end reads from Bowtie2 alignment (.SAM) using HTseq-count program based on annotation file from http://bacteria.ensembl.org. (Escherichia_coli_str_k_12_substr_mg1655.GCA_000005845.2.24.gtf).

Table S5: Mercury exposed DEGs. Fold-change (*worksheet 1*) and $\log_2$ fold-change (*worksheet 2*) values and false discovery rate (FDR) values as determined by baySeq analysis for DEGs under mercury exposed conditions and "n.s." designates expression that was not significantly different from unexposed culture at the same time point (> 1% FDR) and/or less than a two fold-change.

Table S6: COGs. Data from Table S5 grouped by COG's 2014 functional category annotation [2] from DOE IMG database (https://img.jgi.doe.gov). Gene ID's and names highlighted in red indicate duplicate entries that are annotated in more than one category.

Table S7: STRING Network Analysis of $HgCl_2$ and PMA up-regulated genes. Up-regulated genes with ≥ 5 fold-change for mercury exposure conditions and all up-regulated genes for unexposed conditions were input into STRING v10.0 (http://string-db.org/) [3]. STRING database algorithm uses list of differentially expressed gene names as input, but does not include the fold-change intensity in analysis. Nodes

represent genes and line thickness indicates greater correlation score. Network maps for mercury exposures are in Figure S8 and unexposed conditions in Figure S10. Positive DEGs present at each time point are clustered for $HgCl_2$, PMA, and unexposed conditions at each time point.

Table S8: Unexposed DEGs. Fold-change (*worksheet 1*) and log_2 fold-change (*worksheet 2*) values and false discovery rate (FDR) values as determined by baySeq analysis for DEGs at each time point compared to time 0 or for sub-intervals. "n.s." indicates that expression was not significantly different from that of the indicated prior time comparison (> 1% FDR) and/or less than a two fold-change.

Table S9: Replication and repair. Genes involved in DNA replication, recombination, degradation, and repair. Green cells indicate positive fold-change and red means negative fold-change in mercurial exposure condition relative to the unexposed culture at the same time point.

Table S10: Transcription and RNA turnover. Green cells indicate positive fold-change and red means negative fold-change in mercury exposure condition relative to the unexposed culture at the same time point.

Table S11: RegulonDB sigma factors. Differential gene expression of sigma factor genes and corresponding regulon genes for each during mercurial exposure. Gene identities are from RegulonDB (http://regulondb.ccg.unam.mx/index.jsp)[4]. Sigma

factors are shown in bold text and fold-change of genes regulated by each is shown in plain text.

Table S12: RegulonDB transcription factors. Differential gene expression of transcription factor genes (*worksheet 1*) and corresponding regulon genes (*worksheet 2*) during mercurial exposure. Gene identities are from RegulonDB (http://regulondb.ccg.unam.mx/index.jsp)[4]. Transcription factors are shown in bold text and fold-change of genes regulated by each is shown in plain text.

Table S13: Heat map figures data. Data that corresponds to heat map figures, including gene ID, gene name, gene description, and $\log_2$ fold-change values for differentially expressed genes under mercury exposed conditions and “0” designates expression that was not significantly different from unexposed culture at the same time point (> 1% FDR) and/or less than a $\log_2$ fold-change of 1.

Table S14: Translation apparatus. Ribosomal protein genes, organized by operon [5]. Fold-change values of differentially expressed regulator genes for each operon and the genes they regulate are indicated. Also indicated are RHg or Hg adducts observations by LC-MS/MS (Zink, et al., in preparation). Differential expression of other translation-related genes organized by function are shown below the r-proteins table.

Table S15: Metal binding. Genes are grouped by metal bound by each protein and were identified based on gene ontology (GO) annotation in UniProtKB database

(http://www.uniprot.org/uniprot/). Green highlighted cells indicate positive fold-change and red means negative fold-change in mercury exposure condition relative to the unexposed culture at the same time point.

Table S16: OxyRS and SoxRS regulons. Genes grouped by regulator for each regulon had been identified by CHiP-exo [6] and EcoCyc annotation [7] as indicated in column A. Genes highlighted in blue are duplicates that are part of both regulons. Green highlighted cells indicate positive fold-change and red means negative fold-change in mercury exposure condition relative to the unexposed culture at the same time point.

Table S17: Delayed response. Genes that were not differentially expressed at 10 min, but were at 30 and 60 min or just 60 min after mercurial exposure. Up-regulated genes only (*worksheet 1*), down-regulated genes only (*worksheet 2*), and combined (*worksheet 3*). Genes grouped by COG category, green highlighted cells indicated positive fold-change, red mean negative fold-change, and “n.s.” indicates expression that was not significantly different from unexposed culture at the same time point.

Supporting Figures:

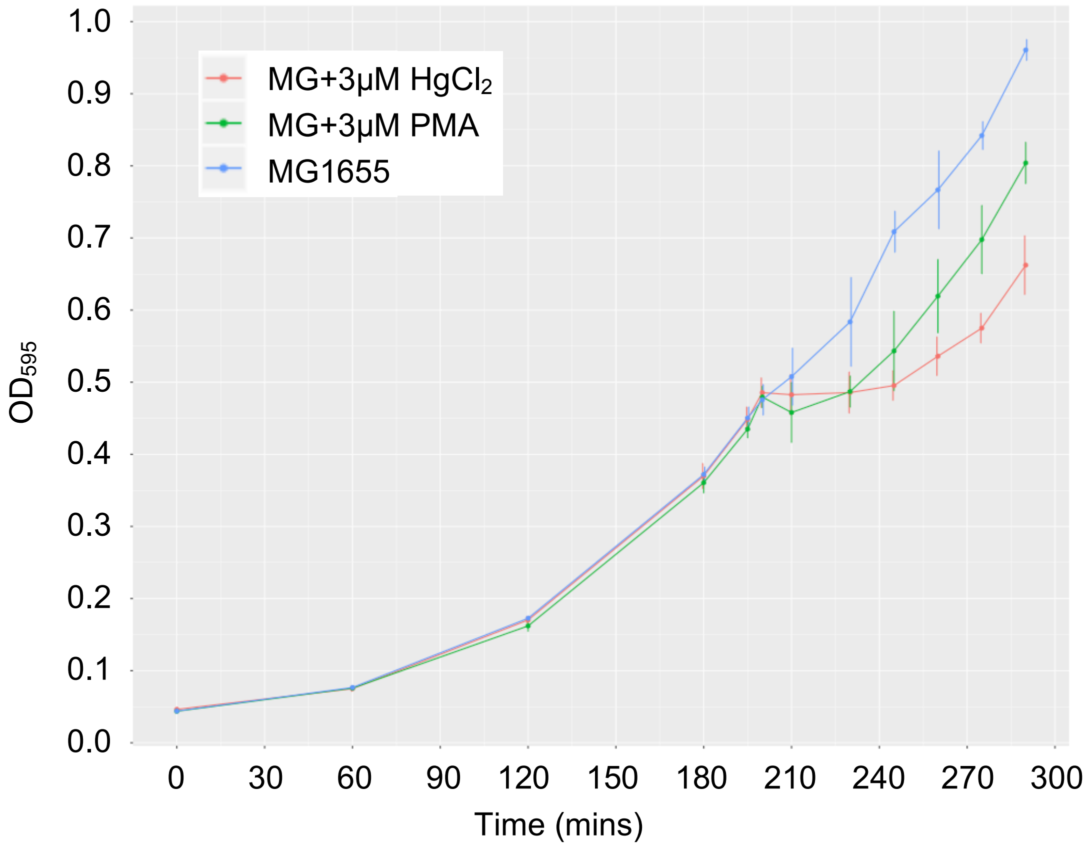

Figure S1: Complete growth curves for all conditions. Average optical density values with standard deviation bars from 3 biological replicate cultures for each condition: MG1655 unexposed culture (blue), MG + 3μM $HgCl_2$ (red), and MG + 3μM PMA (green). Samples for RNA-seq were collected at 200 (t0), 210 (t10), 230 (t30), and 260 (t60) minute time points following exposure to mercury.

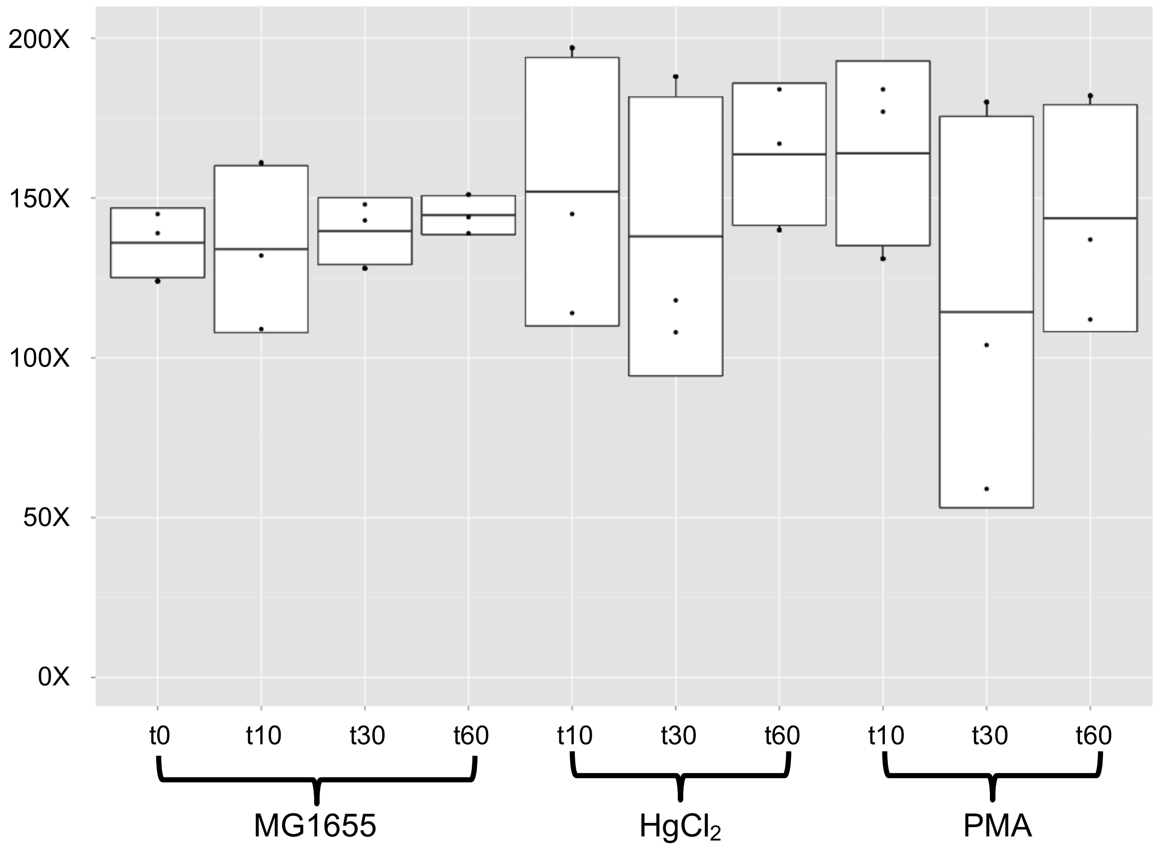

Figure S2: Genome coverage for each condition. The y-axis is the Mapped Reads Genome Coverage for 3 biological replicate libraries at each condition based on the formula: (number of paired-end reads mapped to genome by Bowtie2) x (read length, 50 bp x 2 for paired-end = 100 bp total) / (genome size, 4.64 Mb). Each box represents upper and lower quantiles with the internal horizontal bar as the median and dots indicate data points.

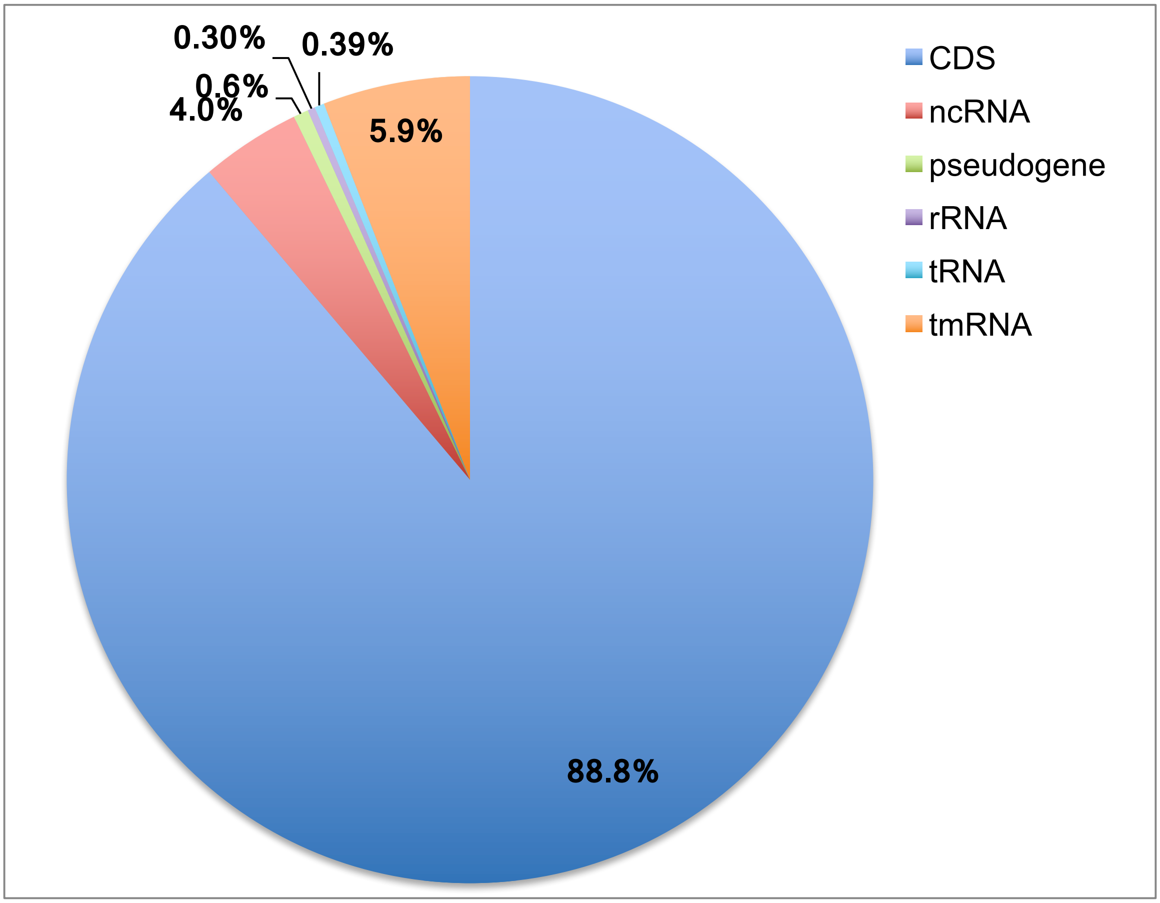

Figure S3: Mapped reads by feature-type for the unexposed control culture averaged across all time points. Percentages were calculated based average raw reads from 3 biological replicates, by taking the sum of reads for each feature type, divided by the total reads for all features, then taking the average across all time points (0,10,30 and 60 mins) for the unexposed control culture. See data in Table S4 details for each condition.

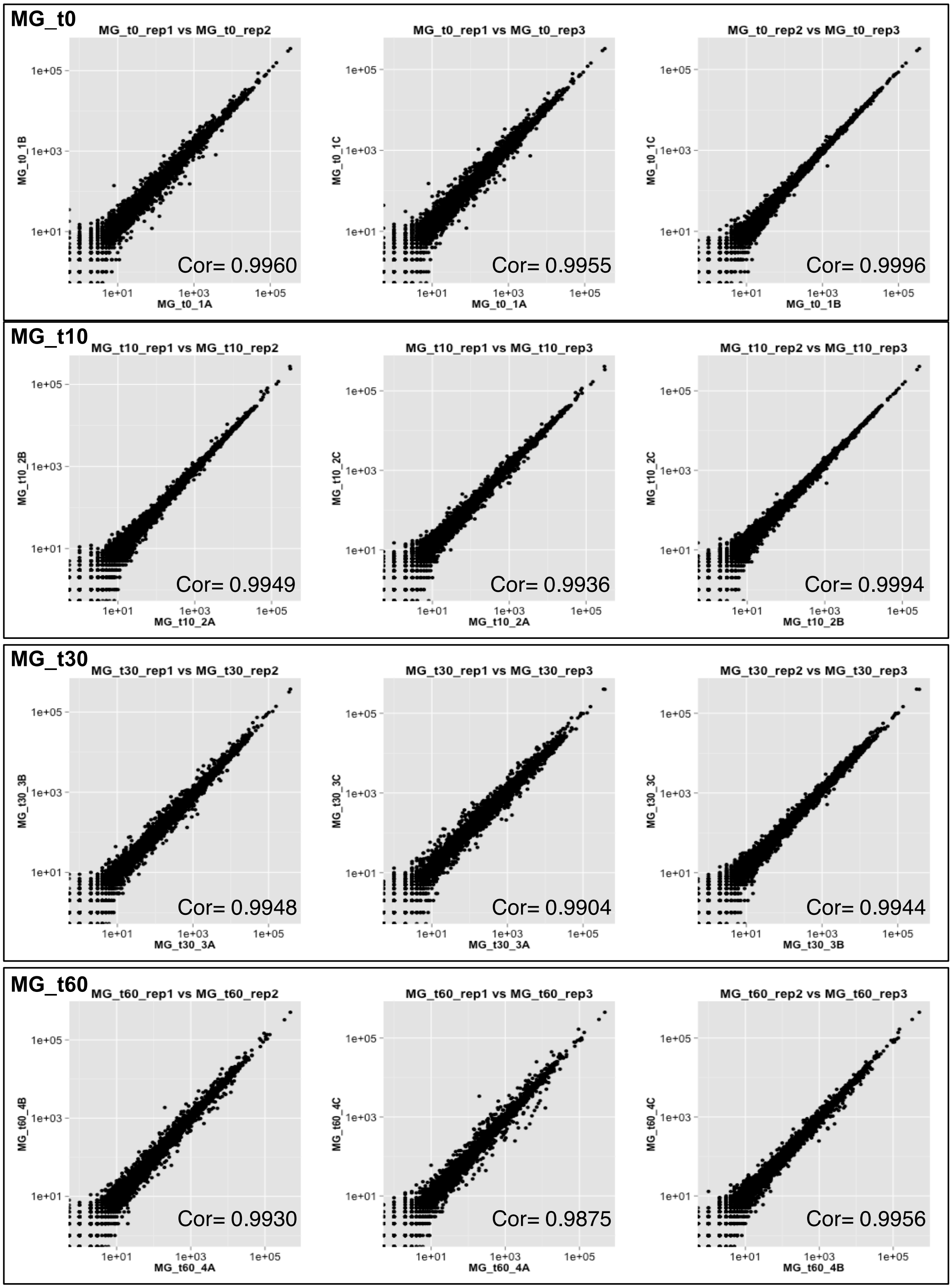
MG_t0
MG_t0_rep1 vs MG_t0_rep2
MG_t0_1B
MG_t0_1A
Cor= 0.9960
MG_t0_rep1 vs MG_t0_rep3
MG_t0_1C
MG_t0_1A
Cor= 0.9955
MG_t0_rep2 vs MG_t0_rep3
MG_t0_1C
MG_t0_1B
Cor= 0.9996
MG_t10
MG_t10_rep1 vs MG_t10_rep2
MG_t10_2B
MG_t10_2A
Cor= 0.9949
MG_t10_rep1 vs MG_t10_rep3
MG_t10_2C
MG_t10_2A
Cor= 0.9936
MG_t10_rep2 vs MG_t10_rep3
MG_t10_2C
MG_t10_2B
Cor= 0.9994
MG_t30
MG_t30_rep1 vs MG_t30_rep2
MG_t30_3B
MG_t30_3A
Cor= 0.9948
MG_t30_rep1 vs MG_t30_rep3
MG_t30_3C
MG_t30_3A
Cor= 0.9904
MG_t30_rep2 vs MG_t30_rep3
MG_t30_3C
MG_t30_3B
Cor= 0.9944
MG_t60
MG_t60_rep1 vs MG_t60_rep2
MG_t60_4B
MG_t60_4A
Cor= 0.9930
MG_t60_rep1 vs MG_t60_rep3
MG_t60_4C
MG_t60_4A
Cor= 0.9875
MG_t60_rep2 vs MG_t60_rep3
MG_t60_4C
MG_t60_4B
Cor= 0.9956
1e+01
1e+03
1e+05

Figure S4: Pearson correlation plots of gene counts in biological replicates for unexposed condition. Pearson correlations were determined by pairwise comparisons of biological replicates (rep1 vs rep2, rep1 vs rep3, rep2 vs rep3) using raw (unnormalized) read counts per gene (with rRNA counts removed) for each condtion examined.

MG+Hg_t10

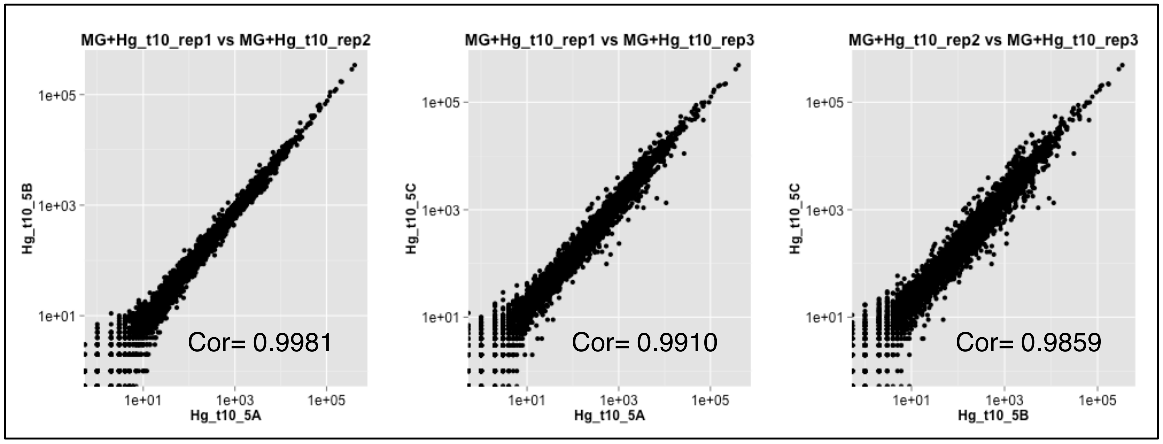

MG+Hg_t30

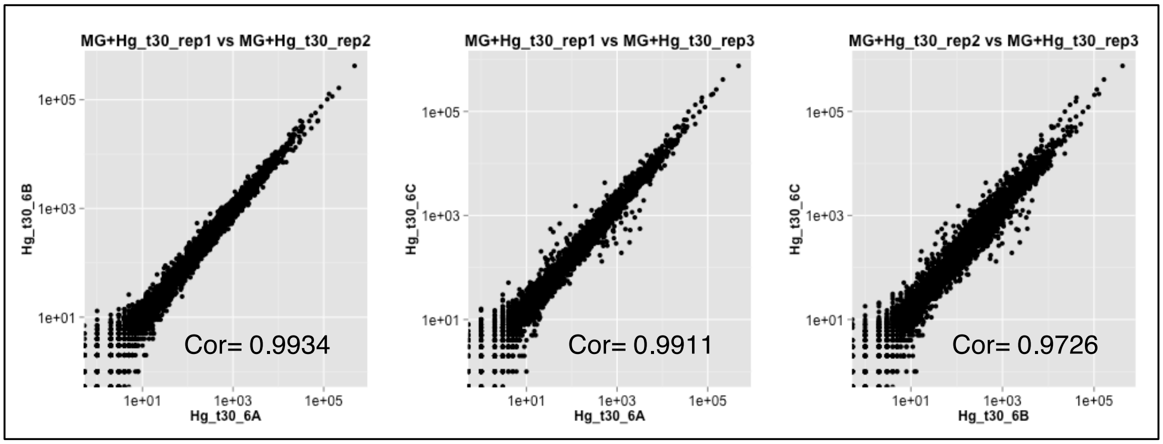

MG+Hg_t60

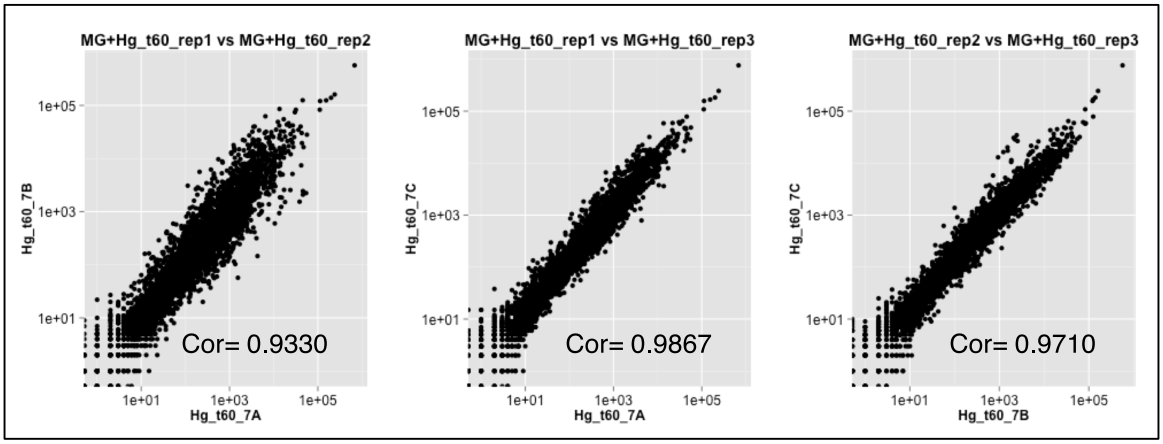

Figure S5: Pearson correlation plots of gene counts in biological replicates for $HgCl_2$ exposed condition. Pearson correlations were determined by pairwise

comparisons of biological replicates (rep1 vs rep2, rep1 vs rep3, rep2 vs rep3) using raw (unnormalized) read counts per gene (with rRNA counts removed) for each condtion examined.

MG+PMA_t10

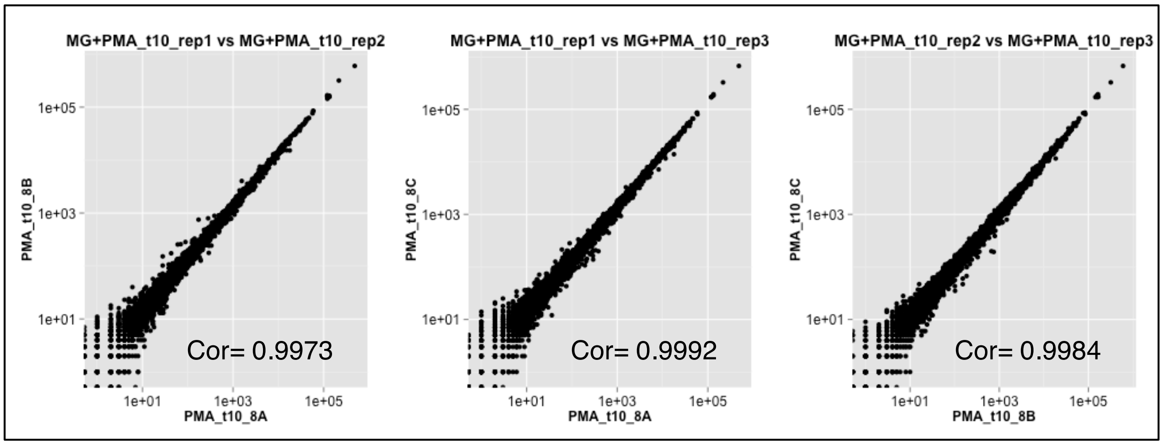

MG+PMA_t30

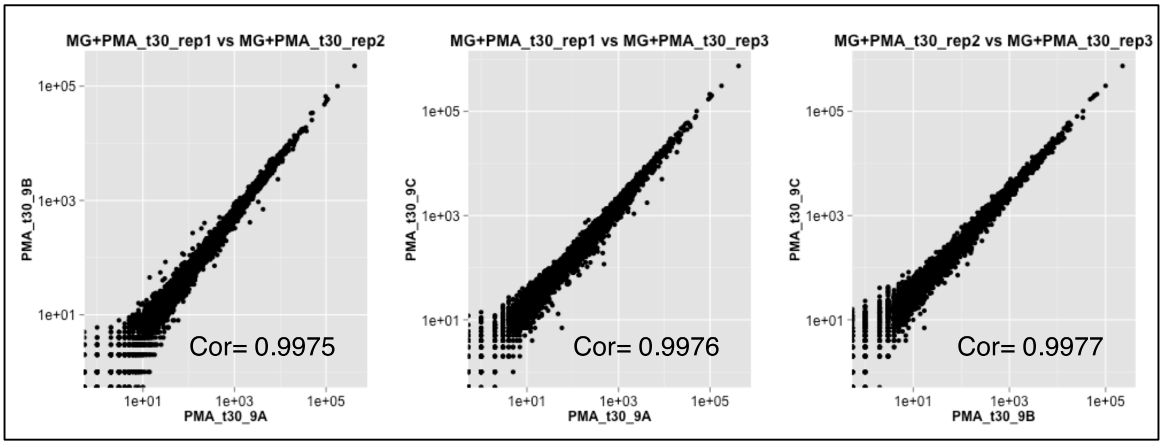

MG+PMA_t60

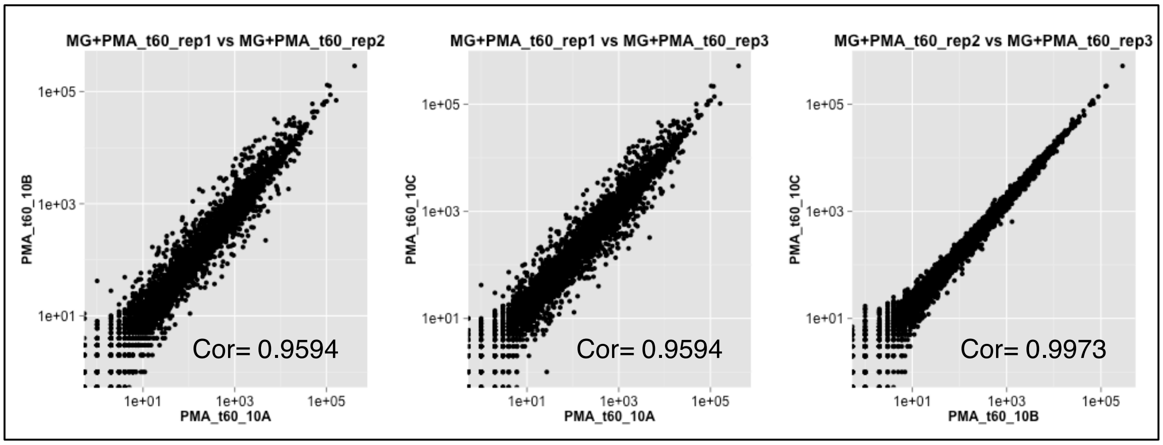

Figure S6: Pearson correlation plots of gene counts in biological replicates for PMA exposed condition. Pearson correlations were determined by pairwise

comparisons of biological replicates (rep1 vs rep2, rep1 vs rep3, rep2 vs rep3) using raw (unnormalized) read counts per gene (with rRNA counts removed) for each condtion examined.

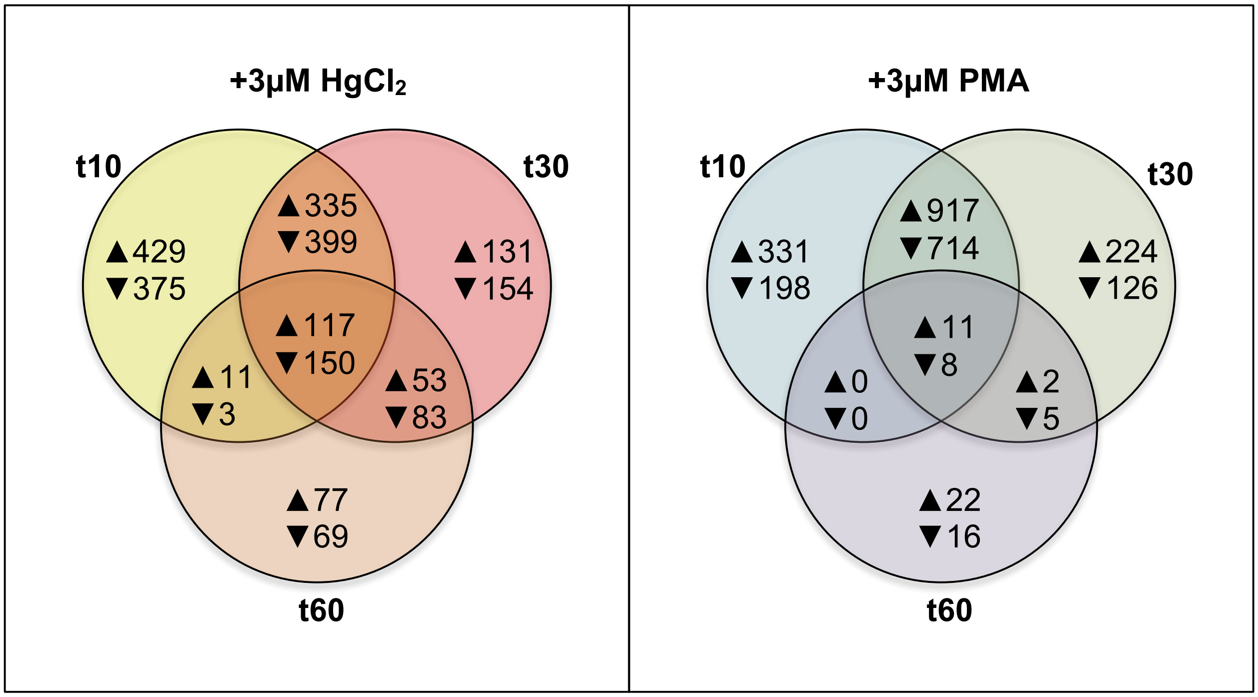

Figure S7: Shared genes at each sampling time for $HgCl_2$ or PMA exposures.

DEGs in outer edge of circles represent unique genes not observed at other time points.

Figure S8: STRING network analysis of up-regulated genes with > 5 fold-change during each mercury exposure condition. GO biological processes categories enriched from network have a FDR of < 5% (see Table S7 for details).

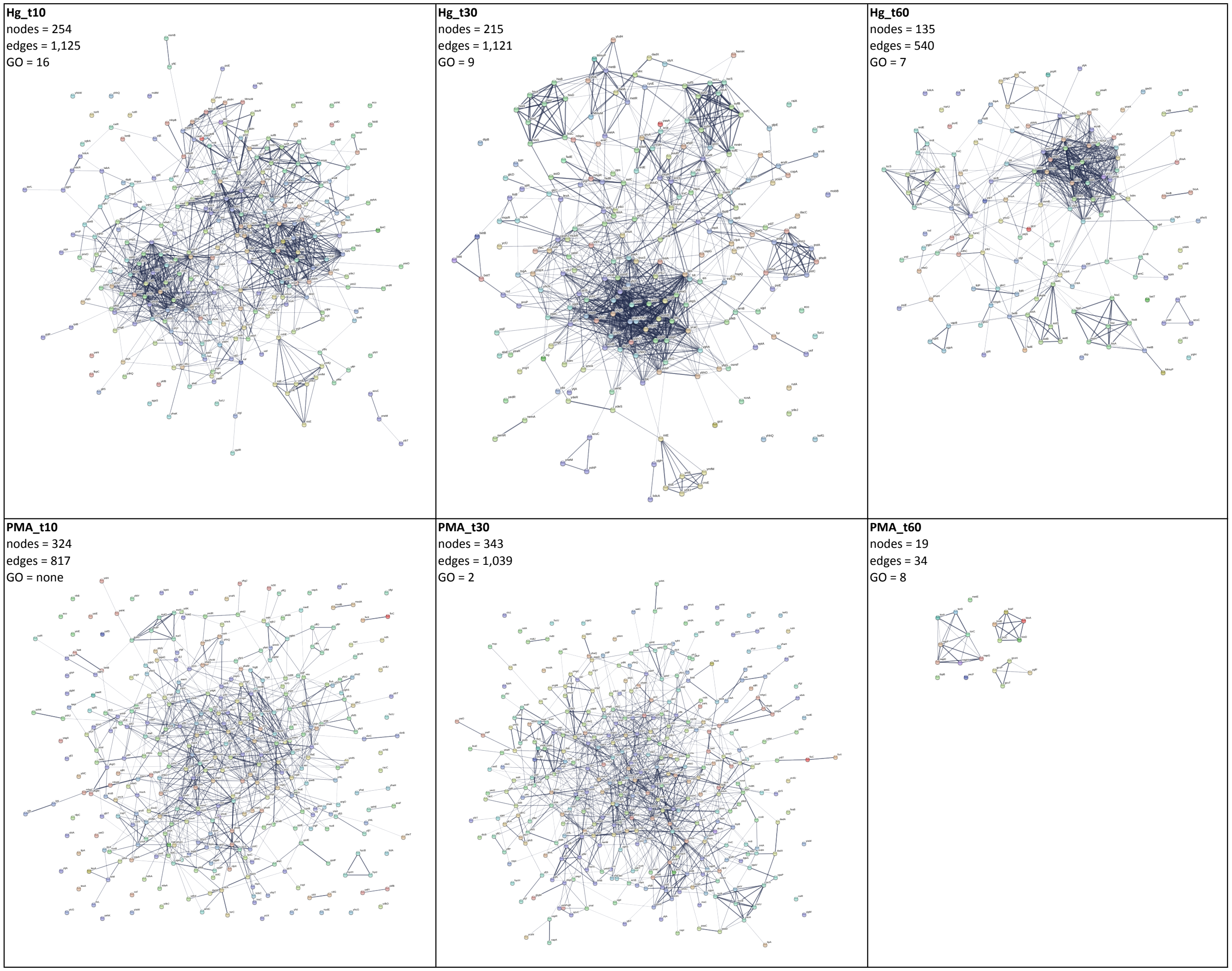

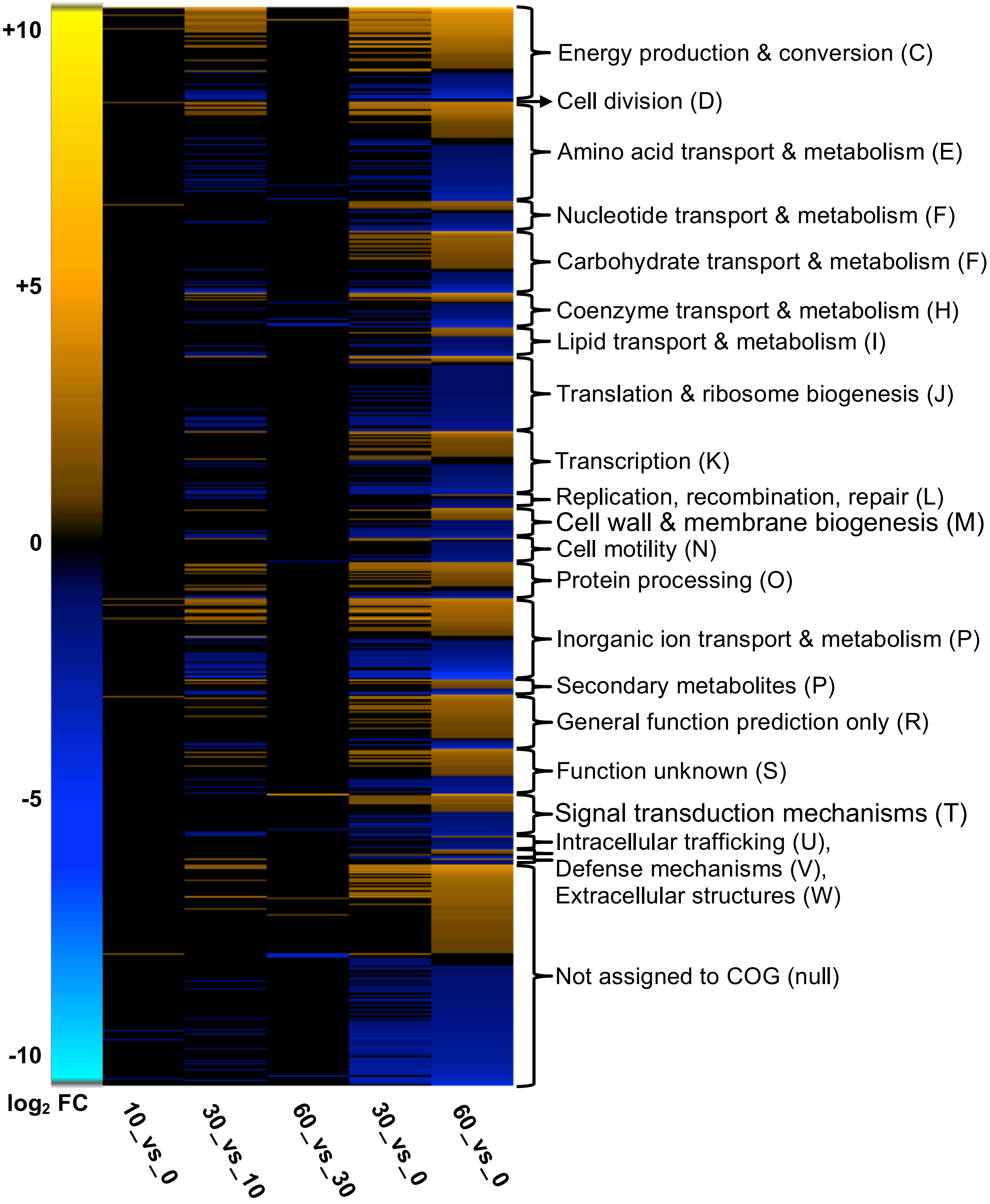

Figure S9: Differentially expressed genes over time in unexposed control culture.

Table sorted highest to lowest for “60_vs_0” column and grouped by COGs 2014

category. Genes with log_2 fold-change ≥ 1 in at least one condition were included and grouped alphabetically by COG category. Conditions: t10_vs_t0 (n=15), t30_vs_t10 (n=195), t60_vs_t30 (n= 16), t30_vs_t0 (n=422), t60_vs_t0 (n=815). Total genes for all conditions = 862 (C=79, D=3, E=87, F=26, G=53, H=31, I=24, J=66, K=55, L=12, M=26, N=20, O=32, P=70, Q=14, R=47, S=39, T=36, U=12, V=8, W=4, X=1, null=193). See Table S8 for details of all genes.

Figure S10: STRING network analysis of all up-regulated genes over time in unexposed culture. GO biological processes categories enriched from network have a FDR of < 5% (see Table S7 for details).

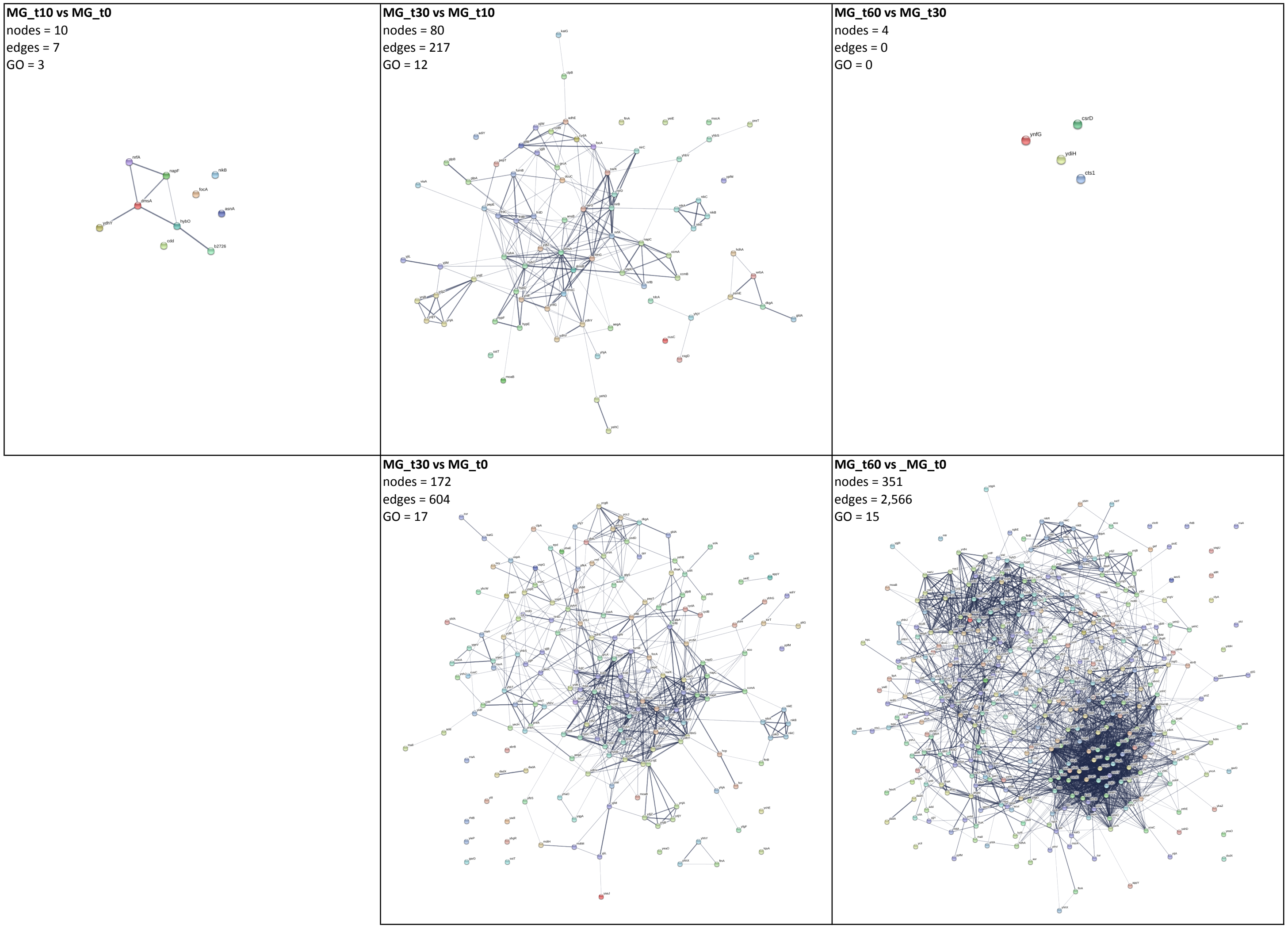

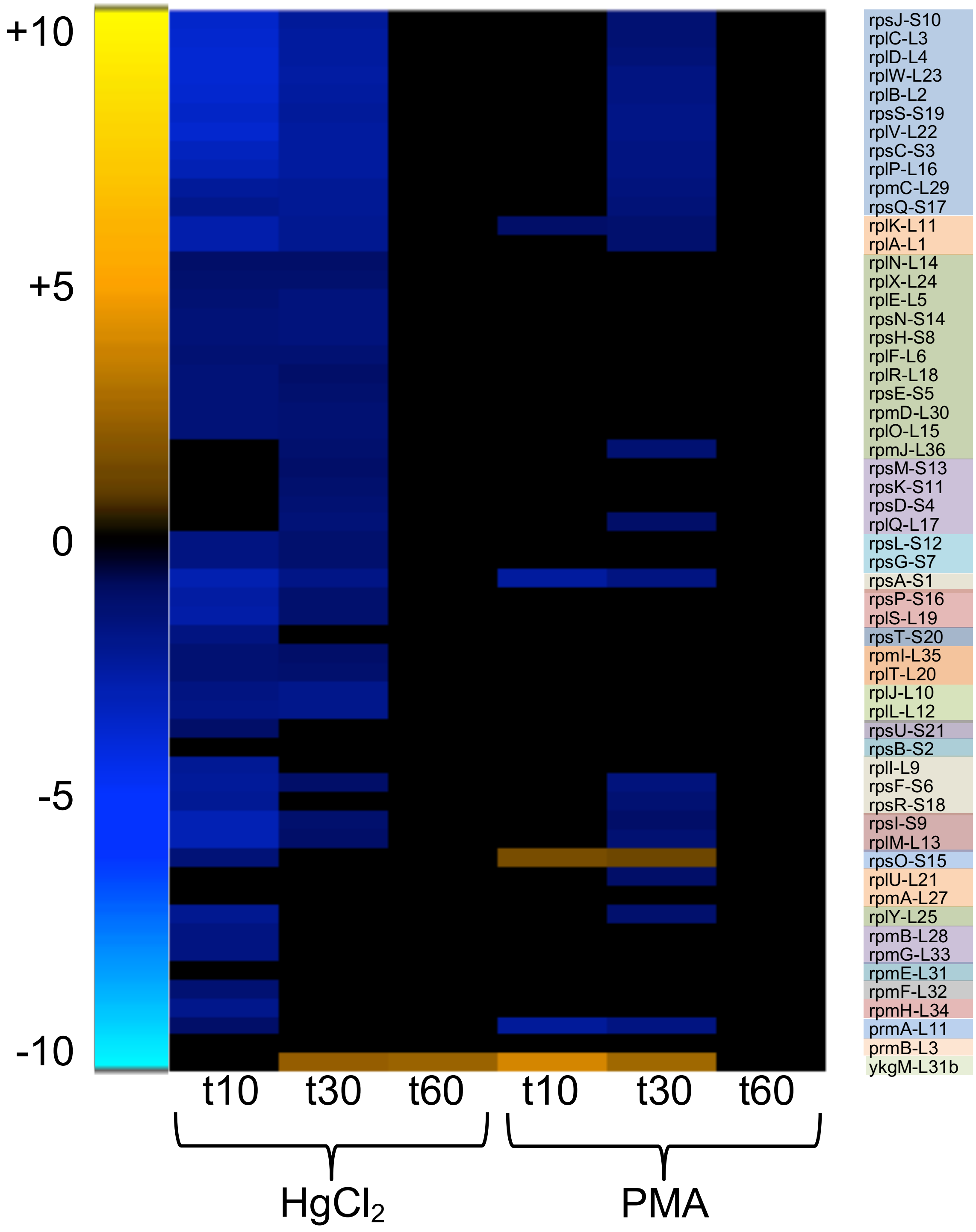

Figure S11 (same as Figure 5): Ribosomal subunit protein genes. Genes are grouped and colored by operon (see Table S14 for details).

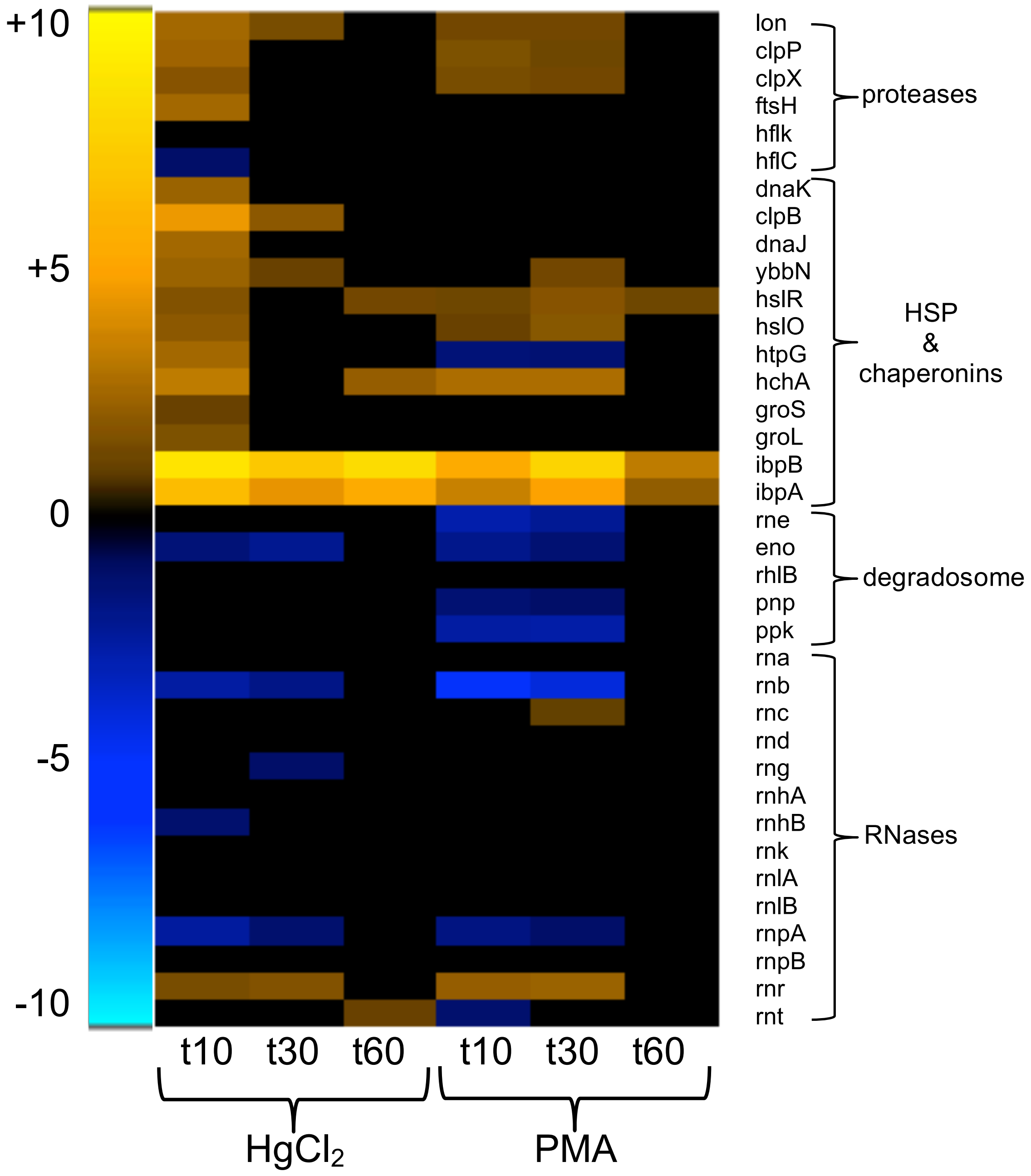

Figure S12 (Figure 6): Proteases, heat shock proteins, degradasome complex and RNases. (See Table S13 for details)

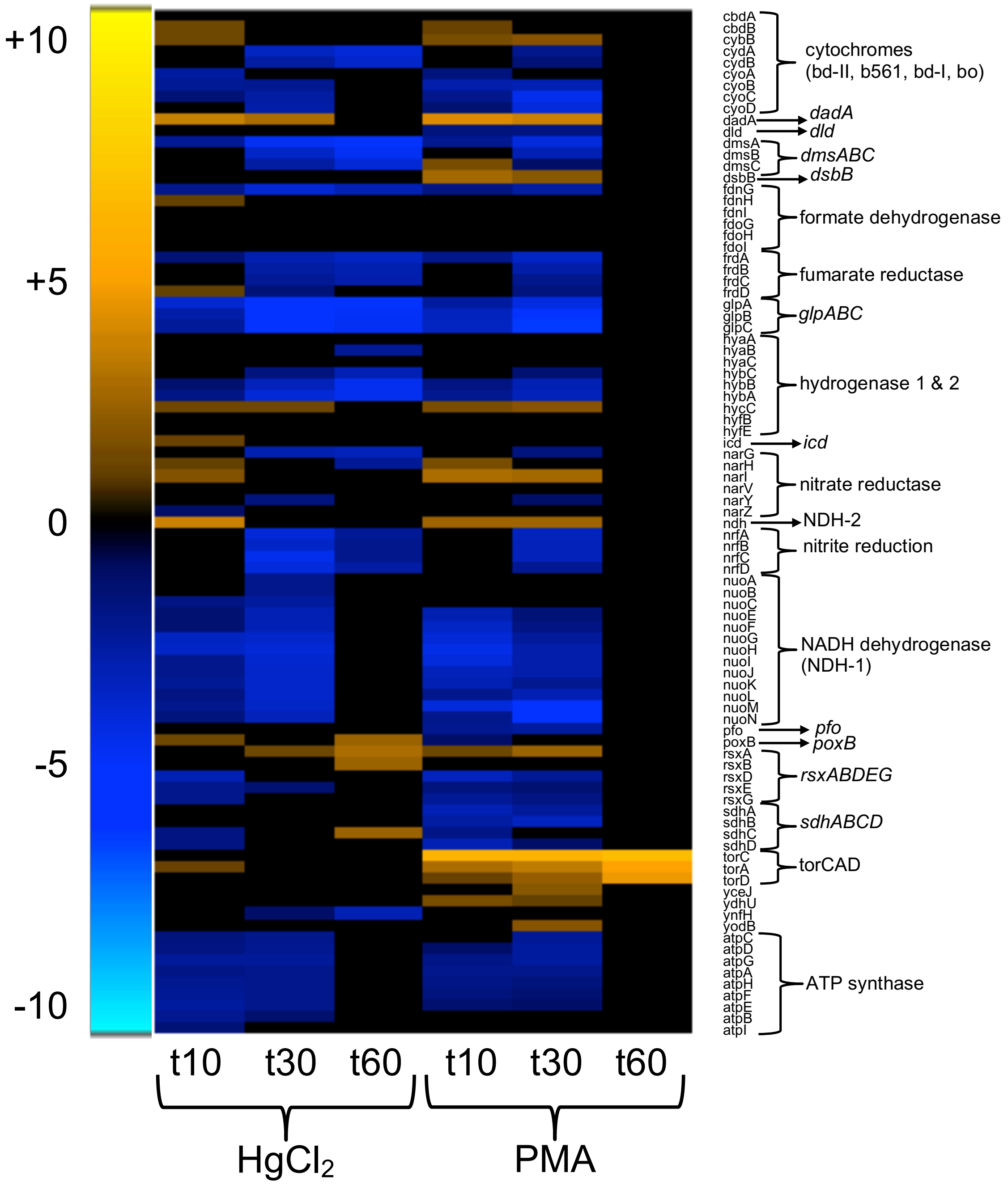

Figure S13 (same as Figure 7): Electron transport chain and ATP-synthase. (See Table S13 for details)

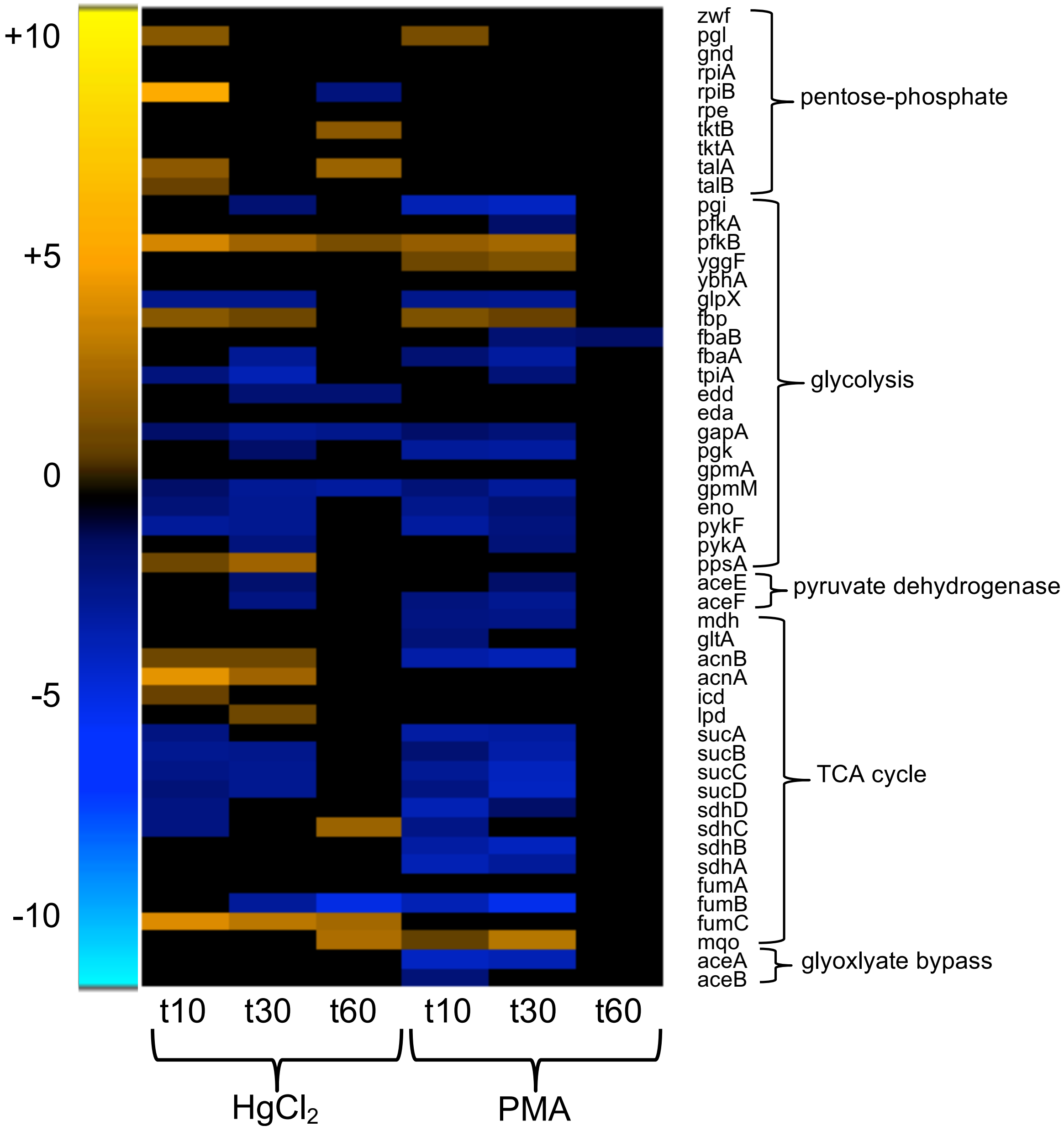

Figure S14: Glucose metabolism. (See Table S13 for details)

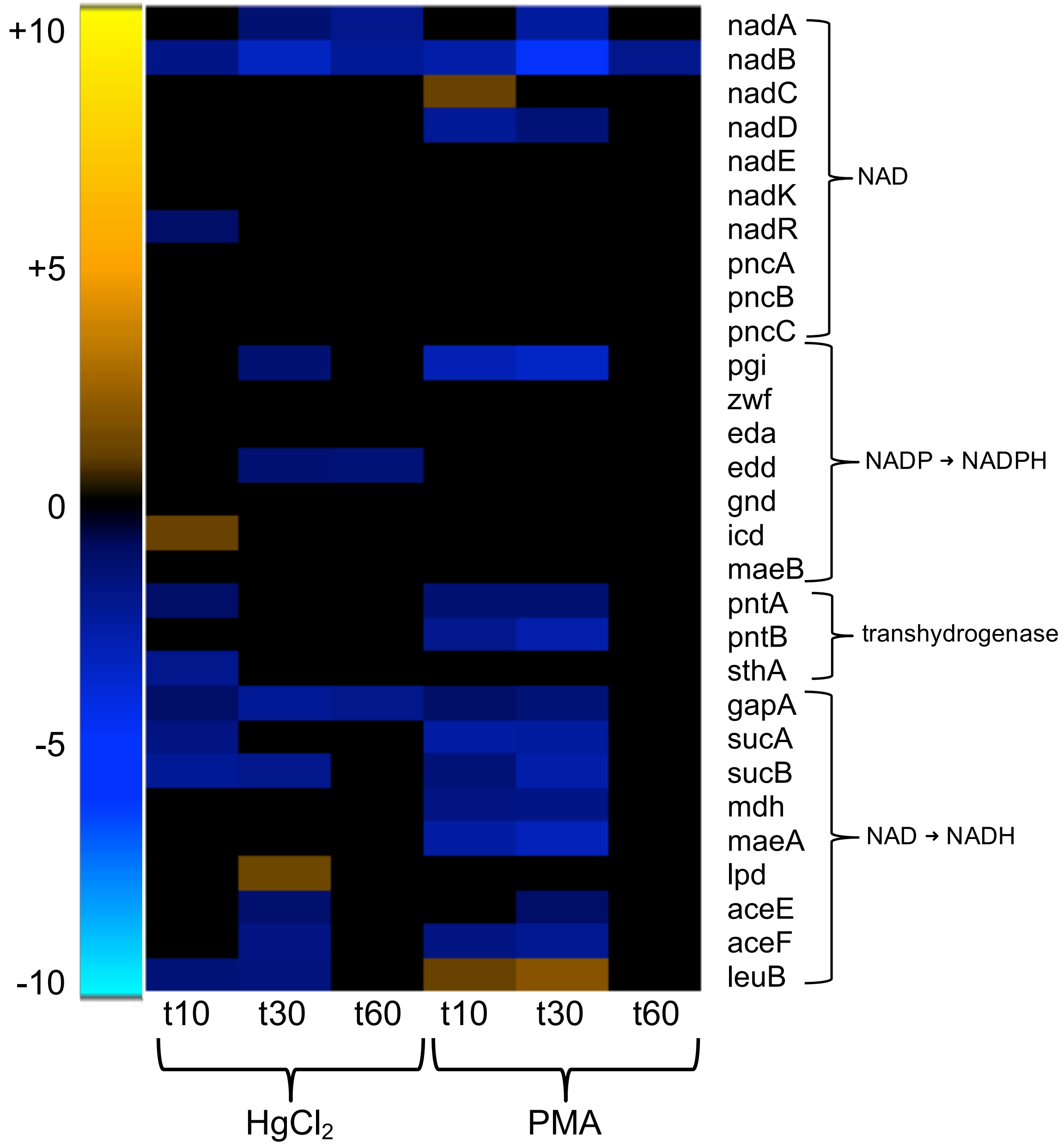

Figure S15: NADH and NADPH synthesis and reduction pathways. (See Table S13 for details)

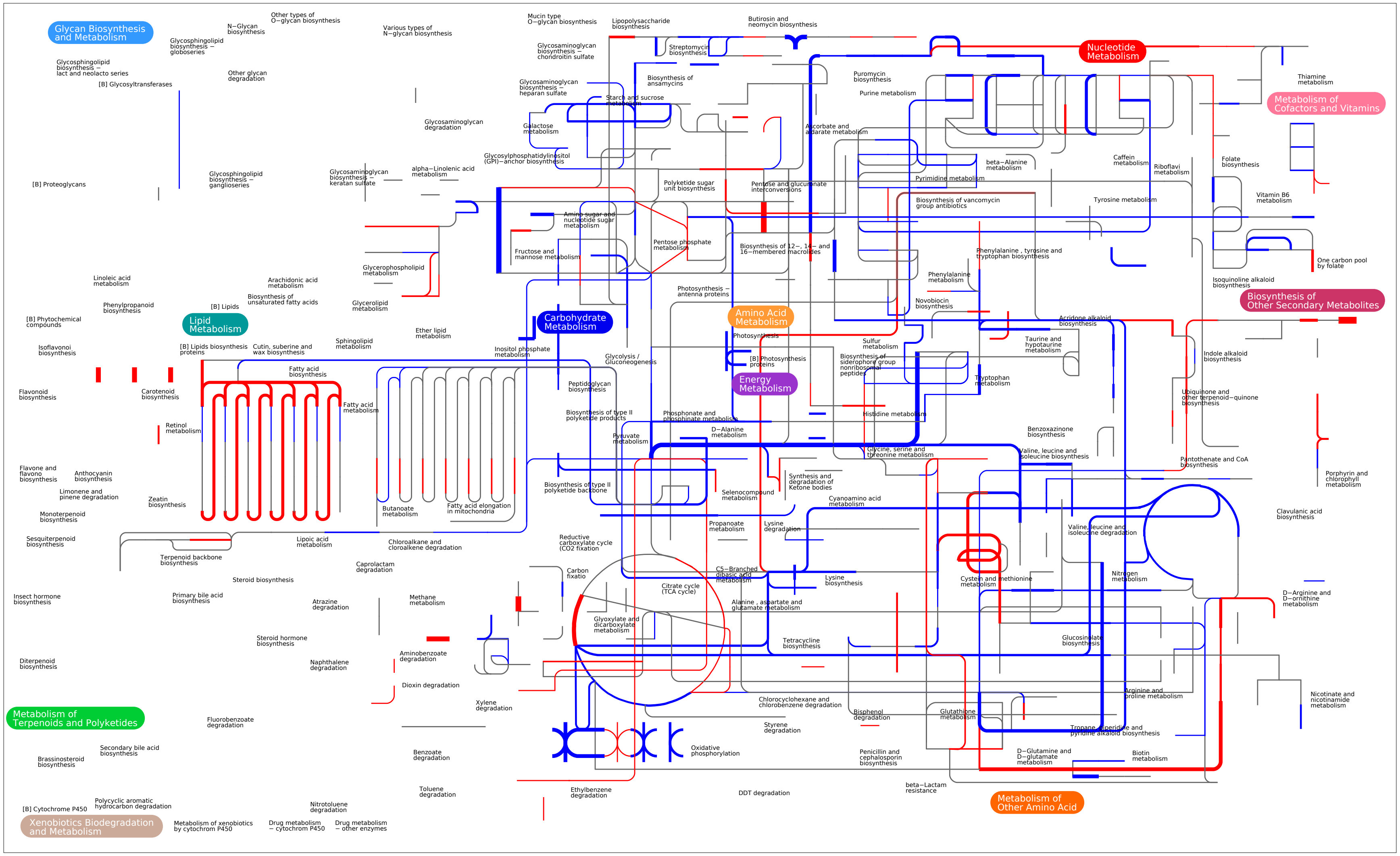
Glycan Biosynthesis and Metabolism
Glycosphingolipid biosynthesis − lact and neolacto series
[B] Glycosyltransferases
Glycosphingolipid biosynthesis − globoseries
N−Glycan biosynthesis
Other types of O−glycan biosynthesis
Other glycan degradation
Various types of N−glycan biosynthesis
Mucin type O−glycan biosynthesis
Lipopolysaccharide biosynthesis
Butirosin and neomycin biosynthesis
Nucleotide Metabolism
Glycosaminoglycan biosynthesis − chondroitin sulfate
Streptomycin biosynthesis
Glycosaminoglycan biosynthesis − heparan sulfate
Biosynthesis of ansamycins
Puromycin biosynthesis
Purine metabolism
Thiamine metabolism
Metabolism of Cofactors and Vitamins
Starch and sucrose metabolism
Glycosaminoglycan degradation
Galactose metabolism
Ascorbate and aldarate metabolism
[B] Proteoglycans
Glycosphingolipid biosynthesis − ganglioseries
Glycosaminoglycan biosynthesis − keratan sulfate
alpha−Linolenic acid metabolism
Glycosylphosphatidylinositol (GPI)−anchor biosynthesis
Polyketide sugar unit biosynthesis
Pentose and glucuronate interconversions
Pyrimidine metabolism
beta−Alanine metabolism
Caffein metabolism
Riboflavin metabolism
Folate biosynthesis
Biosynthesis of vancomycin group antibiotics
Tyrosine metabolism
Vitamin B6 metabolism
Amino sugar and nucleotide sugar metabolism
Fructose and mannose metabolism
Pentose phosphate metabolism
Biosynthesis of 12−, 14− and 16−membered macrolides
Phenylalanine , tyrosine and tryptophan biosynthesis
One carbon pool by folate
Linoleic acid metabolism
Arachidonic acid metabolism
Glycerophospholipid metabolism
Phenylalanine metabolism
Isoquinoline alkaloid biosynthesis
Biosynthesis of unsaturated fatty acids
Photosynthesis − antenna proteins
Novobiocin biosynthesis
Biosynthesis of Other Secondary Metabolites
Phenylpropanoid biosynthesis
[B] Lipids
Glycerolipid metabolism
Amino Acid Metabolism
Acridone alkaloid biosynthesis
[B] Phytochemical compounds
Lipid Metabolism
Ether lipid metabolism
Carbohydrate Metabolism
Photosynthesis
Sulfur metabolism
Taurine and hypotaurine metabolism
Isoflavonoid biosynthesis
[B] Lipids biosynthesis proteins
Cutin, suberine and wax biosynthesis
Sphingolipid metabolism
Inositol phosphate metabolism
Glycolysis / Gluconeogenesis
[B] Photosynthesis proteins
Biosynthesis of siderophore group nonribosomal peptides
Indole alkaloid biosynthesis
Fatty acid biosynthesis
Energy Metabolism
Tryptophan metabolism
Flavonoid biosynthesis
Carotenoid biosynthesis
Fatty acid metabolism
Peptidoglycan biosynthesis
Ubiquinone and other terpenoid−quinone biosynthesis
Biosynthesis of type II polyketide products
Phosphonate and phosphinate metabolism
Histidine metabolism
Retinol metabolism
D−Alanine metabolism
Benzoxazinone biosynthesis
Pyruvate metabolism
Glycine, serine and threonine metabolism
Valine, leucine and isoleucine biosynthesis
Pantothenate and CoA biosynthesis
Flavone and flavonol biosynthesis
Anthocyanin biosynthesis
Biosynthesis of type II polyketide backbone
Synthesis and degradation of Ketone bodies
Porphyrin and chlorophyll metabolism
Limonene and pinene degradation
Selenocompound metabolism
Cyanoamino acid metabolism
Zeatin biosynthesis
Butanoate metabolism
Fatty acid elongation in mitochondria
Clavulanic acid biosynthesis
Monoterpenoid biosynthesis
Propanoate metabolism
Lysine degradation
Valine, leucine and isoleucine degradation
Sesquiterpenoid biosynthesis
Lipoic acid metabolism
Chloroalkane and chloroalkene degradation
Reductive carboxylate cycle (CO2 fixation)
Terpenoid backbone biosynthesis
Caprolactam degradation
Carbon fixation
C5−Branched dibasic acid metabolism
Lysine biosynthesis
Cystein and methionine metabolism
Nitrogen metabolism
Steroid biosynthesis
Citrate cycle (TCA cycle)
D−Arginine and D−ornithine metabolism
Insect hormone biosynthesis
Primary bile acid biosynthesis
Atrazine degradation
Methane metabolism
Alanine , aspartate and glutamate metabolism
Glyoxylate and dicarboxylate metabolism
Steroid hormone biosynthesis
Tetracycline biosynthesis
Glucosinolate biosynthesis
Aminobenzoate degradation
Diterpenoid biosynthesis
Naphthalene degradation
Dioxin degradation
Arginine and proline metabolism
Nicotinate and nicotinamide metabolism
Metabolism of Terpenoids and Polyketides
Xylene degradation
Chlorocyclohexane and chlorobenzene degradation
Fluorobenzoate degradation
Bisphenol degradation
Glutathione metabolism
Tropane, piperidine and pyridine alkaloid biosynthesis
Styrene degradation
Secondary bile acid biosynthesis
Oxidative phosphorylation
Brassinosteroid biosynthesis
Benzoate degradation
Penicillin and cephalosporin biosynthesis
D−Glutamine and D−glutamate metabolism
Biotin metabolism
Toluene degradation
Ethylbenzene degradation
DDT degradation
beta−Lactam resistance
Polycyclic aromatic hydrocarbon degradation
[B] Cytochrome P450
Nitrotoluene degradation
Metabolism of Other Amino Acid
Xenobiotics Biodegradation and Metabolism
Metabolism of xenobiotics by cytochrom P450
Drug metabolism − cytochrom P450
Drug metabolism − other enzymes

Figure S16: iPath of *E. coli* K12 MG1655 metabolic KEGG map during $HgCl_2$ exposure at 10 min. Generated using iPath2.0 [8]. Up-regulated genes are red, down-regulated genes are blue, and increased line thickness indicates greater fold-change.

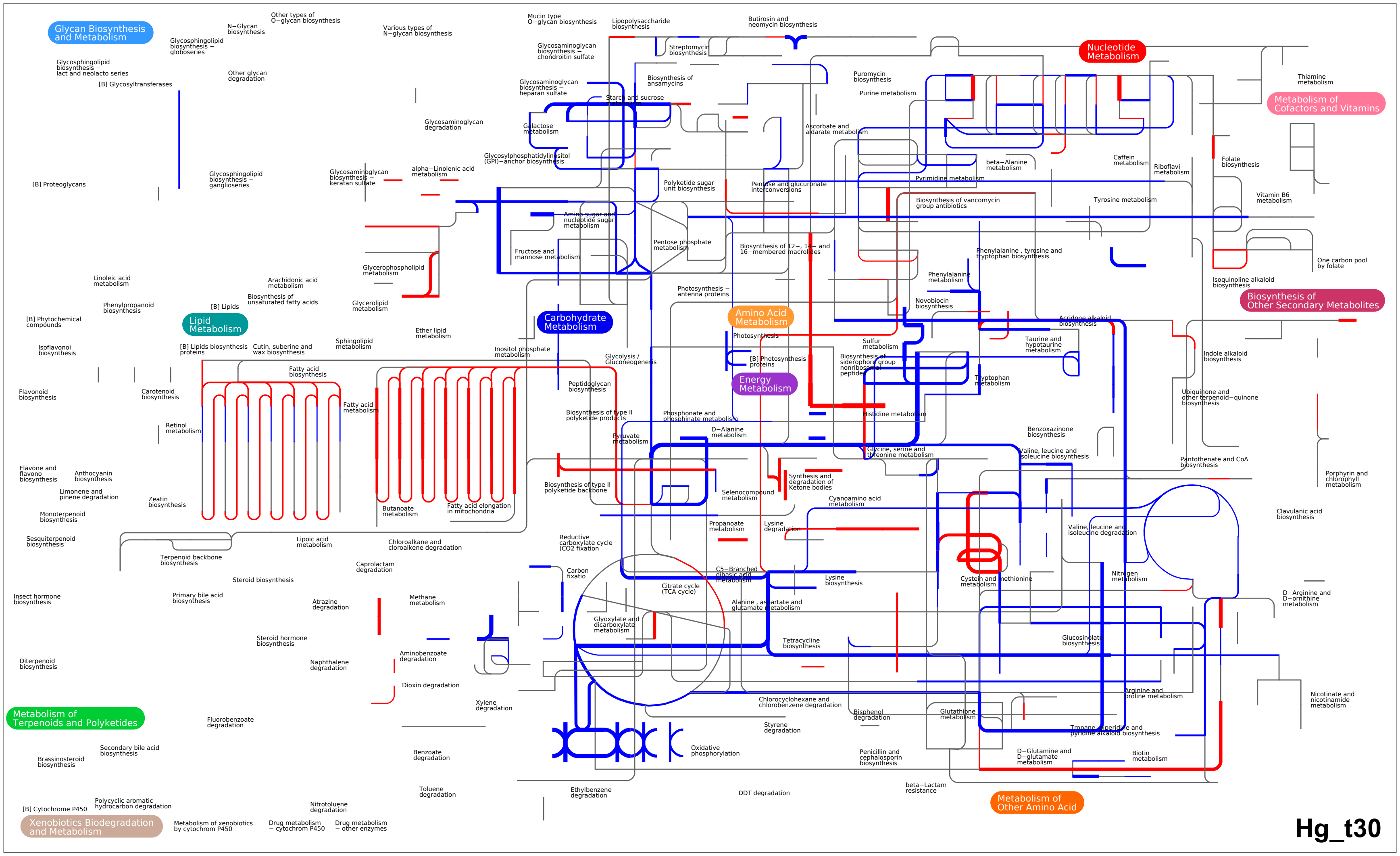

Glycan Biosynthesis and Metabolism
Carbohydrate Metabolism
Lipid Metabolism
Nucleotide Metabolism
Amino Acid Metabolism
Energy Metabolism
Metabolism of Cofactors and Vitamins
Biosynthesis of Other Secondary Metabolites
Metabolism of Other Amino Acid
Metabolism of Terpenoids and Polyketides
Xenobiotics Biodegradation and Metabolism
Glycosphingolipid biosynthesis − lact and neolacto series
Glycosphingolipid biosynthesis − globoseries
Glycosphingolipid biosynthesis − ganglioseries
N−Glycan biosynthesis
Other types of O−glycan biosynthesis
Various types of N−glycan biosynthesis
Other glycan degradation
[B] Glycosyltransferases
[B] Proteoglycans
Mucin type O−glycan biosynthesis
Lipopolysaccharide biosynthesis
Butirosin and neomycin biosynthesis
Streptomycin biosynthesis
Glycosaminoglycan biosynthesis − chondroitin sulfate
Glycosaminoglycan biosynthesis − heparan sulfate
Glycosaminoglycan biosynthesis − keratan sulfate
Glycosaminoglycan degradation
Biosynthesis of ansamycins
Starch and sucrose metabolism
Galactose metabolism
Glycosylphosphatidylinositol (GPI)−anchor biosynthesis
alpha−Linolenic acid metabolism
Amino sugar and nucleotide sugar metabolism
Fructose and mannose metabolism
Polyketide sugar unit biosynthesis
Pentose phosphate metabolism
Pentose and glucuronate interconversions
Ascorbate and aldarate metabolism
Puromycin biosynthesis
Purine metabolism
Pyrimidine metabolism
beta−Alanine metabolism
Caffeine metabolism
Riboflavin metabolism
Thiamine metabolism
Folate biosynthesis
Vitamin B6 metabolism
One carbon pool by folate
Biosynthesis of vancomycin group antibiotics
Tyrosine metabolism
Biosynthesis of 12−, 14− and 16−membered macrolides
Phenylalanine , tyrosine and tryptophan biosynthesis
Phenylalanine metabolism
Novobiocin biosynthesis
Isoquinoline alkaloid biosynthesis
Acridone alkaloid biosynthesis
Taurine and hypotaurine metabolism
Indole alkaloid biosynthesis
Sulfur metabolism
Biosynthesis of siderophore group nonribosomal peptides
Tryptophan metabolism
Ubiquinone and other terpenoid−quinone biosynthesis
Histidine metabolism
Benzoxazinone biosynthesis
Valine, leucine and isoleucine biosynthesis
Pantothenate and CoA biosynthesis
Porphyrin and chlorophyll metabolism
Glycine, serine and threonine metabolism
Linoleic acid metabolism
Arachidonic acid metabolism
Biosynthesis of unsaturated fatty acids
Glycerophospholipid metabolism
Glycerolipid metabolism
Phenylpropanoid biosynthesis
[B] Lipids
[B] Phytochemical compounds
Ether lipid metabolism
Isoflavonoid biosynthesis
[B] Lipids biosynthesis proteins
Cutin, suberine and wax biosynthesis
Sphingolipid metabolism
Inositol phosphate metabolism
Glycolysis / Gluconeogenesis
Photosynthesis − antenna proteins
Photosynthesis
[B] Photosynthesis proteins
Flavonoid biosynthesis
Carotenoid biosynthesis
Fatty acid biosynthesis
Fatty acid metabolism
Peptidoglycan biosynthesis
Biosynthesis of type II polyketide products
Phosphonate and phosphinate metabolism
Retinol metabolism
Pyruvate metabolism
D−Alanine metabolism
Flavone and flavonol biosynthesis
Anthocyanin biosynthesis
Limonene and pinene degradation
Zeatin biosynthesis
Monoterpenoid biosynthesis
Butanoate metabolism
Fatty acid elongation in mitochondria
Biosynthesis of type II polyketide backbone
Selenocompound metabolism
Synthesis and degradation of Ketone bodies
Cyanoamino acid metabolism
Propanoate metabolism
Lysine degradation
Valine, leucine and isoleucine degradation
Clavulanic acid biosynthesis
Sesquiterpenoid biosynthesis
Lipoic acid metabolism
Chloroalkane and chloroalkene degradation
Reductive carboxylate cycle (CO2 fixation)
Terpenoid backbone biosynthesis
Caprolactam degradation
Carbon fixation
C5−Branched dibasic acid metabolism
Lysine biosynthesis
Cystein and methionine metabolism
Nitrogen metabolism
Steroid biosynthesis
Citrate cycle (TCA cycle)
D−Arginine and D−ornithine metabolism
Insect hormone biosynthesis
Primary bile acid biosynthesis
Atrazine degradation
Methane metabolism
Glyoxylate and dicarboxylate metabolism
Alanine , aspartate and glutamate metabolism
Steroid hormone biosynthesis
Tetracycline biosynthesis
Glucosinolate biosynthesis
Aminobenzoate degradation
Diterpenoid biosynthesis
Naphthalene degradation
Dioxin degradation
Arginine and proline metabolism
Nicotinate and nicotinamide metabolism
Xylene degradation
Chlorocyclohexane and chlorobenzene degradation
Bisphenol degradation
Glutathione metabolism
Fluorobenzoate degradation
Tropane, piperidine and pyridine alkaloid biosynthesis
Secondary bile acid biosynthesis
Styrene degradation
Brassinosteroid biosynthesis
Benzoate degradation
Oxidative phosphorylation
Penicillin and cephalosporin biosynthesis
D−Glutamine and D−glutamate metabolism
Biotin metabolism
Toluene degradation
Ethylbenzene degradation
DDT degradation
beta−Lactam resistance
Polycyclic aromatic hydrocarbon degradation
[B] Cytochrome P450
Nitrotoluene degradation
Metabolism of xenobiotics by cytochrom P450
Drug metabolism − cytochrom P450
Drug metabolism − other enzymes
Hg_t30

Figure S17: iPath of *E. coli* K12 MG1655 metabolic KEGG map during $HgCl_2$ exposure at 30 min. Generated using iPath2.0 [8]. Up-regulated genes are red, down-regulated genes are blue, and increased line thickness indicates greater fold-change.

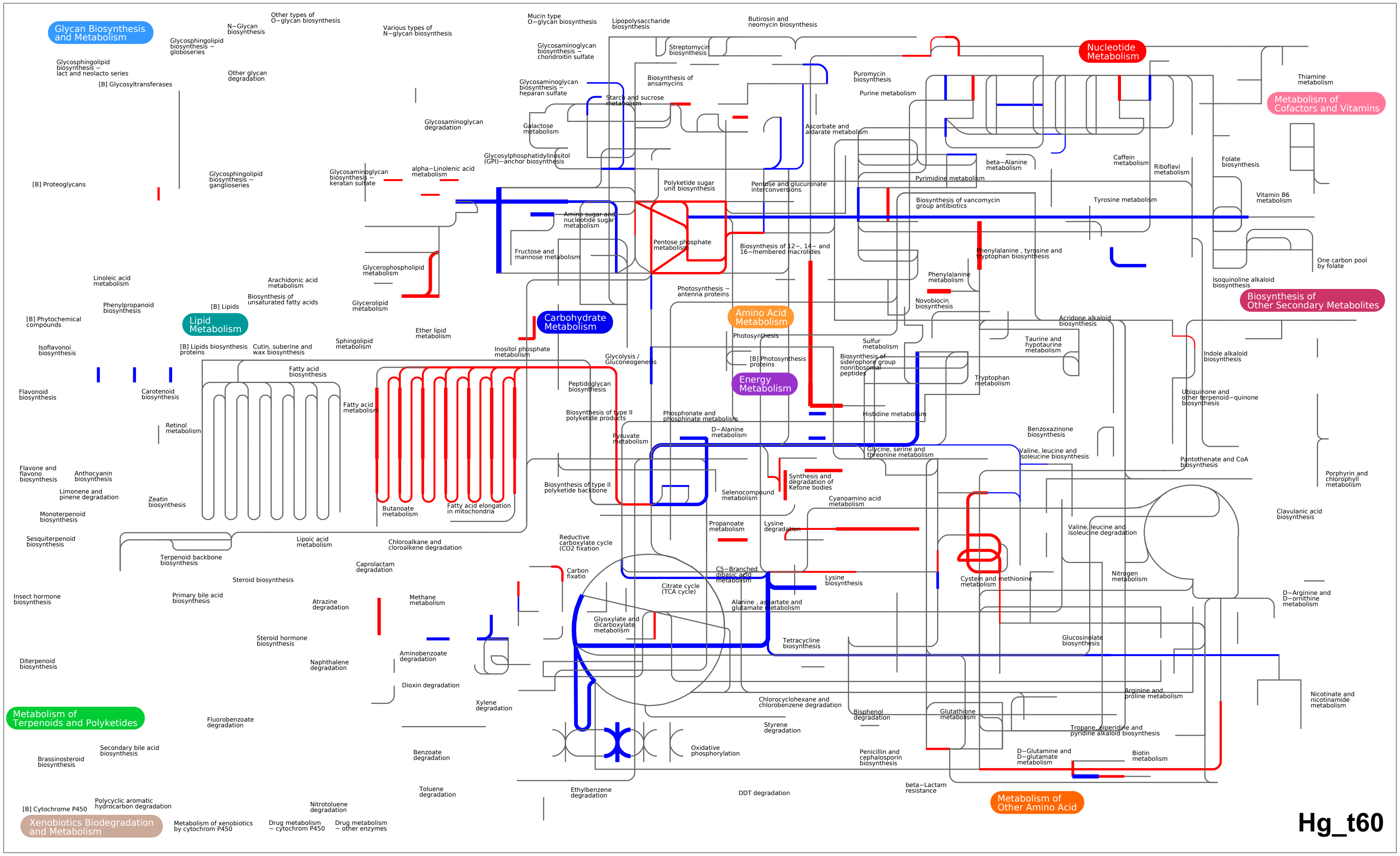
Glycan Biosynthesis and Metabolism
N−Glycan biosynthesis
Other types of O−glycan biosynthesis
Various types of N−glycan biosynthesis
Mucin type O−glycan biosynthesis
Lipopolysaccharide biosynthesis
Butirosin and neomycin biosynthesis
Glycosphingolipid biosynthesis − globoseries
Glycosphingolipid biosynthesis − lact and neolacto series
[B] Glycosyltransferases
Other glycan degradation
Glycosaminoglycan biosynthesis − chondroitin sulfate
Streptomycin biosynthesis
Nucleotide Metabolism
Glycosaminoglycan biosynthesis − heparan sulfate
Biosynthesis of ansamycins
Puromycin biosynthesis
Thiamine metabolism
Starch and sucrose metabolism
Purine metabolism
Metabolism of Cofactors and Vitamins
Glycosaminoglycan degradation
Galactose metabolism
Ascorbate and aldarate metabolism
Glycosylphosphatidylinositol (GPI)−anchor biosynthesis
beta−Alanine metabolism
Caffein metabolism
Riboflavi metabolism
Folate biosynthesis
[B] Proteoglycans
Glycosphingolipid biosynthesis − ganglioseries
Glycosaminoglycan biosynthesis − keratan sulfate
alpha−Linolenic acid metabolism
Polyketide sugar unit biosynthesis
Pentose and glucuronate interconversions
Pyrimidine metabolism
Vitamin B6 metabolism
Biosynthesis of vancomycin group antibiotics
Tyrosine metabolism
Amino sugar and nucleotide sugar metabolism
Fructose and mannose metabolism
Pentose phosphate metabolism
Biosynthesis of 12−, 14− and 16−membered macrolides
Phenylalanine , tyrosine and tryptophan biosynthesis
One carbon pool by folate
Linoleic acid metabolism
Arachidonic acid metabolism
Glycerophospholipid metabolism
Phenylalanine metabolism
Isoquinoline alkaloid biosynthesis
Biosynthesis of unsaturated fatty acids
Photosynthesis − antenna proteins
Novobiocin biosynthesis
Biosynthesis of Other Secondary Metabolites
Phenylpropanoid biosynthesis
[B] Lipids
Glycerolipid metabolism
Amino Acid Metabolism
Acridone alkaloid biosynthesis
[B] Phytochemical compounds
Lipid Metabolism
Carbohydrate Metabolism
Ether lipid metabolism
Photosynthesis
Sulfur metabolism
Taurine and hypotaurine metabolism
Isoflavonoi biosynthesis
[B] Lipids biosynthesis proteins
Cutin, suberine and wax biosynthesis
Sphingolipid metabolism
Inositol phosphate metabolism
Glycolysis / Gluconeogenesis
[B] Photosynthesis proteins
Biosynthesis of siderophore group nonribosomal peptides
Indole alkaloid biosynthesis
Fatty acid biosynthesis
Energy Metabolism
Tryptophan metabolism
Flavonoid biosynthesis
Carotenoid biosynthesis
Peptidoglycan biosynthesis
Fatty acid metabolism
Ubiquinone and other terpenoid−quinone biosynthesis
Biosynthesis of type II polyketide products
Phosphonate and phosphinate metabolism
Histidine metabolism
Retinol metabolism
D−Alanine metabolism
Benzoxazinone biosynthesis
Pyruvate metabolism
Glycine, serine and threonine metabolism
Valine, leucine and isoleucine biosynthesis
Pantothenate and CoA biosynthesis
Flavone and flavonol biosynthesis
Anthocyanin biosynthesis
Synthesis and degradation of Ketone bodies
Porphyrin and chlorophyll metabolism
Biosynthesis of type II polyketide backbone
Selenocompound metabolism
Limonene and pinene degradation
Zeatin biosynthesis
Butanoate metabolism
Fatty acid elongation in mitochondria
Cyanoamino acid metabolism
Clavulanic acid biosynthesis
Monoterpenoid biosynthesis
Propanoate metabolism
Lysine degradation
Valine, leucine and isoleucine degradation
Sesquiterpenoid biosynthesis
Lipoic acid metabolism
Chloroalkane and cloroalkene degradation
Reductive carboxylate cycle (CO2 fixation)
Terpenoid backbone biosynthesis
Caprolactam degradation
Carbon fixatio
C5−Branched dibasic acid metabolism
Lysine biosynthesis
Cystein and methionine metabolism
Nitrogen metabolism
Steroid biosynthesis
Citrate cycle (TCA cycle)
Insect hormone biosynthesis
Primary bile acid biosynthesis
Atrazine degradation
Methane metabolism
Alanine , aspartate and glutamate metabolism
D−Arginine and D−ornithine metabolism
Glyoxylate and dicarboxylate metabolism
Steroid hormone biosynthesis
Tetracycline biosynthesis
Glucosinolate biosynthesis
Aminobenzoate degradation
Diterpenoid biosynthesis
Naphthalene degradation
Dioxin degradation
Arginine and proline metabolism
Nicotinate and nicotinamide metabolism
Xylene degradation
Chlorocyclohexane and chlorobenzene degradation
Metabolism of Terpenoids and Polyketides
Fluorobenzoate degradation
Bisphenol degradation
Glutathione metabolism
Styrene degradation
Tropane, piperidine and pyridine alkaloid biosynthesis
Secondary bile acid biosynthesis
Oxidative phosphorylation
Brassinosteroid biosynthesis
Benzoate degradation
Penicillin and cephalosporin biosynthesis
D−Glutamine and D−glutamate metabolism
Biotin metabolism
Toluene degradation
Ethylbenzene degradation
DDT degradation
beta−Lactam resistance
Polycyclic aromatic hydrocarbon degradation
[B] Cytochrome P450
Nitrotoluene degradation
Metabolism of Other Amino Acid
Xenobiotics Biodegradation and Metabolism
Metabolism of xenobiotics by cytochrom P450
Drug metabolism − cytochrom P450
Drug metabolism − other enzymes
Hg_t60

Figure S18: iPath of *E. coli* K12 MG1655 metabolic KEGG map during $HgCl_2$ exposure at 60 min. Generated using iPath2.0 [8]. Up-regulated genes are red, down-regulated genes are blue, and increased line thickness indicates greater fold-change.

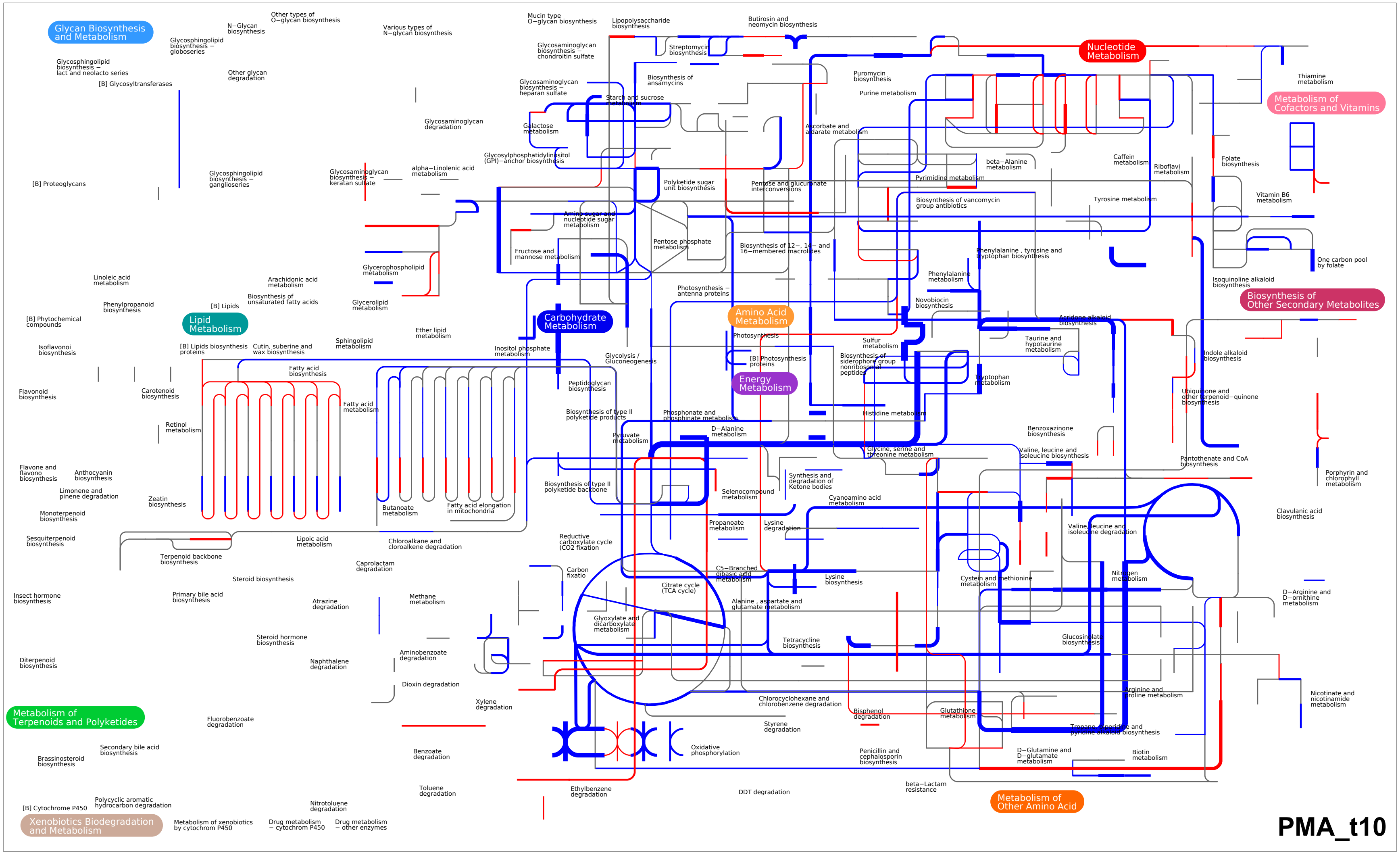
Glycan Biosynthesis and Metabolism
Nucleotide Metabolism
Metabolism of Cofactors and Vitamins
Biosynthesis of Other Secondary Metabolites
Lipid Metabolism
Carbohydrate Metabolism
Amino Acid Metabolism
Energy Metabolism
Metabolism of Terpenoids and Polyketides
Xenobiotics Biodegradation and Metabolism
Metabolism of Other Amino Acid
PMA_t10
N−Glycan biosynthesis
Other types of O−glycan biosynthesis
Various types of N−glycan biosynthesis
Mucin type O−glycan biosynthesis
Lipopolysaccharide biosynthesis
Butirosin and neomycin biosynthesis
Glycosphingolipid biosynthesis − globoseries
Glycosphingolipid biosynthesis − lact and neolacto series
[B] Glycosyltransferases
Other glycan degradation
Glycosaminoglycan biosynthesis − chondroitin sulfate
Streptomycin biosynthesis
Glycosaminoglycan biosynthesis − heparan sulfate
Biosynthesis of ansamycins
Puromycin biosynthesis
Purine metabolism
Thiamine metabolism
Starch and sucrose metabolism
Glycosaminoglycan degradation
Galactose metabolism
Ascorbate and aldarate metabolism
Glycosylphosphatidylinositol (GPI)−anchor biosynthesis
beta−Alanine metabolism
Caffeine metabolism
Riboflavin metabolism
Folate biosynthesis
Pyrimidine metabolism
[B] Proteoglycans
Glycosphingolipid biosynthesis − ganglioseries
Glycosaminoglycan biosynthesis − keratan sulfate
alpha−Linolenic acid metabolism
Polyketide sugar unit biosynthesis
Pentose and glucuronate interconversions
Biosynthesis of vancomycin group antibiotics
Tyrosine metabolism
Vitamin B6 metabolism
Amino sugar and nucleotide sugar metabolism
Fructose and mannose metabolism
Pentose phosphate metabolism
Biosynthesis of 12−, 14− and 16−membered macrolides
Phenylalanine , tyrosine and tryptophan biosynthesis
One carbon pool by folate
Glycerophospholipid metabolism
Linoleic acid metabolism
Arachidonic acid metabolism
Phenylalanine metabolism
Isoquinoline alkaloid biosynthesis
Photosynthesis − antenna proteins
Phenylpropanoid biosynthesis
[B] Lipids
Biosynthesis of unsaturated fatty acids
Glycerolipid metabolism
Novobiocin biosynthesis
[B] Phytochemical compounds
Ether lipid metabolism
Acridone alkaloid biosynthesis
Photosynthesis
Sulfur metabolism
Taurine and hypotaurine metabolism
Isoflavonoid biosynthesis
[B] Lipids biosynthesis proteins
Cutin, suberine and wax biosynthesis
Sphingolipid metabolism
Inositol phosphate metabolism
Glycolysis / Gluconeogenesis
[B] Photosynthesis proteins
Biosynthesis of siderophore group nonribosomal peptides
Indole alkaloid biosynthesis
Fatty acid biosynthesis
Tryptophan metabolism
Flavonoid biosynthesis
Carotenoid biosynthesis
Peptidoglycan biosynthesis
Ubiquinone and other terpenoid−quinone biosynthesis
Fatty acid metabolism
Biosynthesis of type II polyketide products
Phosphonate and phosphinate metabolism
Histidine metabolism
Retinol metabolism
D−Alanine metabolism
Benzoxazinone biosynthesis
Pyruvate metabolism
Glycine, serine and threonine metabolism
Valine, leucine and isoleucine biosynthesis
Pantothenate and CoA biosynthesis
Flavone and flavonol biosynthesis
Anthocyanin biosynthesis
Porphyrin and chlorophyll metabolism
Biosynthesis of type II polyketide backbone
Synthesis and degradation of Ketone bodies
Limonene and pinene degradation
Selenocompound metabolism
Zeatin biosynthesis
Butanoate metabolism
Fatty acid elongation in mitochondria
Cyanoamino acid metabolism
Clavulanic acid biosynthesis
Monoterpenoid biosynthesis
Propanoate metabolism
Lysine degradation
Valine, leucine and isoleucine degradation
Sesquiterpenoid biosynthesis
Lipoic acid metabolism
Chloroalkane and cloroalkene degradation
Reductive carboxylate cycle (CO2 fixation
Terpenoid backbone biosynthesis
Caprolactam degradation
Carbon fixatio
C5−Branched dibasic acid metabolism
Nitrogen metabolism
Steroid biosynthesis
Lysine biosynthesis
Cystein and methionine metabolism
Citrate cycle (TCA cycle)
Insect hormone biosynthesis
Primary bile acid biosynthesis
Methane metabolism
D−Arginine and D−ornithine metabolism
Atrazine degradation
Alanine , aspartate and glutamate metabolism
Glyoxylate and dicarboxylate metabolism
Steroid hormone biosynthesis
Tetracycline biosynthesis
Glucosinolate biosynthesis
Aminobenzoate degradation
Diterpenoid biosynthesis
Naphthalene degradation
Dioxin degradation
Arginine and proline metabolism
Nicotinate and nicotinamide metabolism
Xylene degradation
Chlorocyclohexane and chlorobenzene degradation
Bisphenol degradation
Glutathione metabolism
Fluorobenzoate degradation
Styrene degradation
Tropane, piperidine and pyridine alkaloid biosynthesis
Secondary bile acid biosynthesis
Oxidative phosphorylation
Penicillin and cephalosporin biosynthesis
D−Glutamine and D−glutamate metabolism
Biotin metabolism
Brassinosteroid biosynthesis
Benzoate degradation
Toluene degradation
Ethylbenzene degradation
DDT degradation
beta−Lactam resistance
Polycyclic aromatic hydrocarbon degradation
Nitrotoluene degradation
[B] Cytochrome P450
Metabolism of xenobiotics by cytochrom P450
Drug metabolism − cytochrom P450
Drug metabolism − other enzymes

Figure S19: iPath of *E. coli* K12 MG1655 metabolic KEGG map during PMA exposure at 10 min. Generated using iPath2.0 [8]. Up-regulated genes are red, down-regulated genes are blue, and increased line thickness indicates greater fold-change.

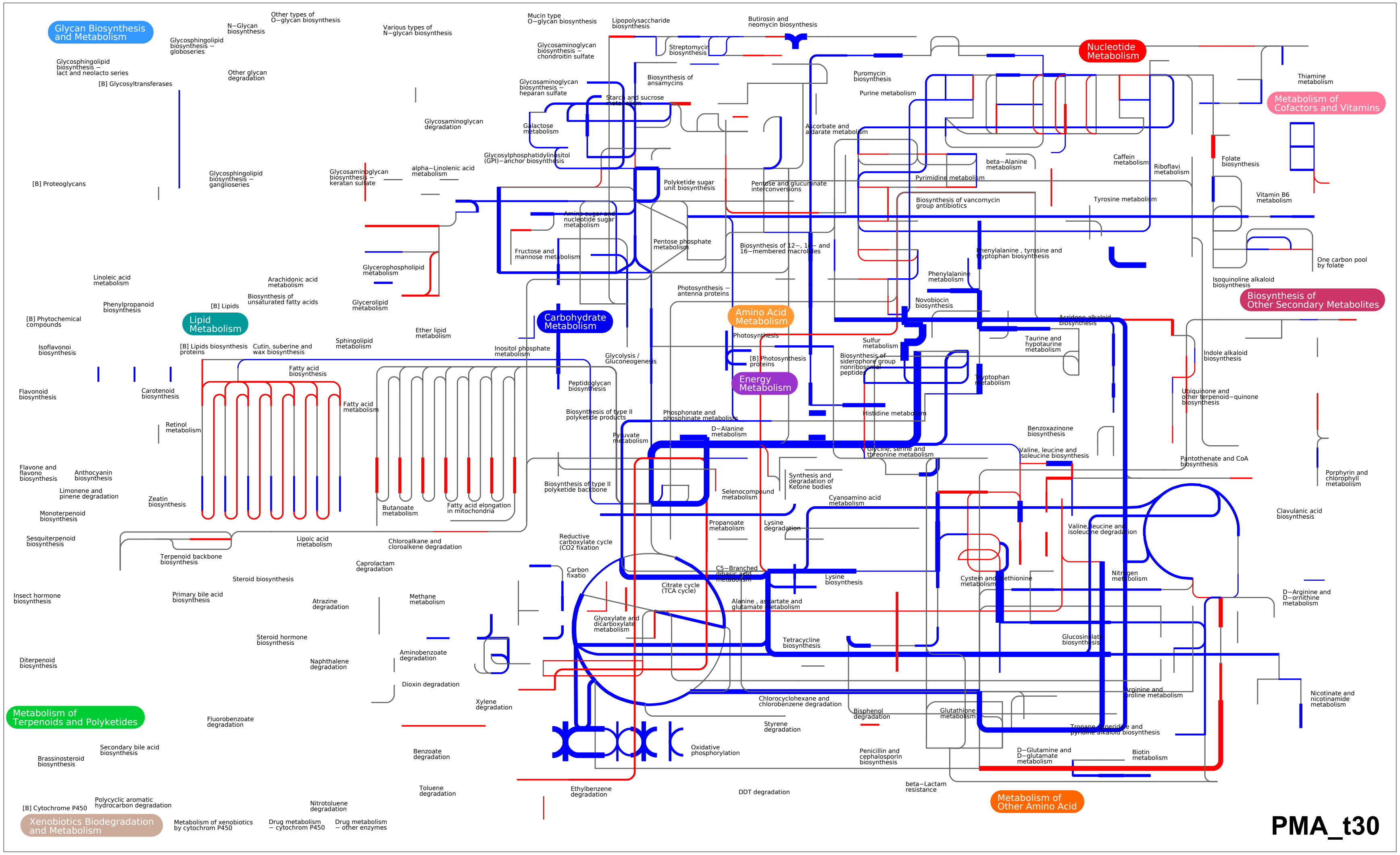
Glycan Biosynthesis and Metabolism
N−Glycan biosynthesis
Other types of O−glycan biosynthesis
Various types of N−glycan biosynthesis
Mucin type O−glycan biosynthesis
Lipopolysaccharide biosynthesis
Butirosin and neomycin biosynthesis
Glycosphingolipid biosynthesis − globoseries
Glycosphingolipid biosynthesis − lact and neolacto series
[B] Glycosyltransferases
Other glycan degradation
Glycosaminoglycan biosynthesis − chondroitin sulfate
Streptomycin biosynthesis
Nucleotide Metabolism
Glycosaminoglycan biosynthesis − heparan sulfate
Biosynthesis of ansamycins
Puromycin biosynthesis
Purine metabolism
Thiamine metabolism
Metabolism of Cofactors and Vitamins
Starch and sucrose metabolism
Glycosaminoglycan degradation
Galactose metabolism
Ascorbate and aldarate metabolism
[B] Proteoglycans
Glycosphingolipid biosynthesis − ganglioseries
Glycosaminoglycan biosynthesis − keratan sulfate
alpha−Linolenic acid metabolism
Glycosylphosphatidylinositol (GPI)−anchor biosynthesis
Polyketide sugar unit biosynthesis
Pentose and glucuronate interconversions
beta−Alanine metabolism
Pyrimidine metabolism
Caffeine metabolism
Riboflavin metabolism
Folate biosynthesis
Biosynthesis of vancomycin group antibiotics
Tyrosine metabolism
Vitamin B6 metabolism
Amino sugar and nucleotide sugar metabolism
Fructose and mannose metabolism
Pentose phosphate pathway
Biosynthesis of 12−, 14− and 16−membered macrolides
Phenylalanine, tyrosine and tryptophan biosynthesis
One carbon pool by folate
Linoleic acid metabolism
Arachidonic acid metabolism
Glycerophospholipid metabolism
Phenylalanine metabolism
Isoquinoline alkaloid biosynthesis
Photosynthesis − antenna proteins
Biosynthesis of Other Secondary Metabolites
Phenylpropanoid biosynthesis
[B] Lipids
Biosynthesis of unsaturated fatty acids
Glycerolipid metabolism
Novobiocin biosynthesis
[B] Phytochemical compounds
Lipid Metabolism
Carbohydrate Metabolism
Amino Acid Metabolism
Acridone alkaloid biosynthesis
Ether lipid metabolism
Photosynthesis
Sulfur metabolism
Taurine and hypotaurine metabolism
Isoflavonoid biosynthesis
[B] Lipids biosynthesis proteins
Cutin, suberine and wax biosynthesis
Sphingolipid metabolism
Inositol phosphate metabolism
Glycolysis / Gluconeogenesis
[B] Photosynthesis proteins
Biosynthesis of siderophore group nonribosomal peptides
Indole alkaloid biosynthesis
Fatty acid biosynthesis
Energy Metabolism
Tryptophan metabolism
Flavonoid biosynthesis
Carotenoid biosynthesis
Peptidoglycan biosynthesis
Ubiquinone and other terpenoid−quinone biosynthesis
Fatty acid metabolism
Biosynthesis of type II polyketide products
Phosphonate and phosphinate metabolism
Histidine metabolism
Retinol metabolism
D−Alanine metabolism
Benzoxazinone biosynthesis
Pyruvate metabolism
Glycine, serine and threonine metabolism
Valine, leucine and isoleucine biosynthesis
Pantothenate and CoA biosynthesis
Flavone and flavonol biosynthesis
Anthocyanin biosynthesis
Porphyrin and chlorophyll metabolism
Biosynthesis of type II polyketide backbone
Synthesis and degradation of Ketone bodies
Limonene and pinene degradation
Zeatin biosynthesis
Butanoate metabolism
Fatty acid elongation in mitochondria
Selenocompound metabolism
Cyanoamino acid metabolism
Clavulanic acid biosynthesis
Monoterpenoid biosynthesis
Propanoate metabolism
Lysine degradation
Valine, leucine and isoleucine degradation
Sesquiterpenoid biosynthesis
Lipoic acid metabolism
Chloroalkane and chloroalkene degradation
Reductive carboxylate cycle (CO2 fixation)
Terpenoid backbone biosynthesis
Caprolactam degradation
Carbon fixation
C5−Branched dibasic acid metabolism
Lysine biosynthesis
Cysteine and methionine metabolism
Nitrogen metabolism
Steroid biosynthesis
Citrate cycle (TCA cycle)
Insect hormone biosynthesis
Primary bile acid biosynthesis
Atrazine degradation
Methane metabolism
Alanine, aspartate and glutamate metabolism
D−Arginine and D−ornithine metabolism
Glyoxylate and dicarboxylate metabolism
Steroid hormone biosynthesis
Tetracycline biosynthesis
Glucosinolate biosynthesis
Diterpenoid biosynthesis
Naphthalene degradation
Aminobenzoate degradation
Dioxin degradation
Arginine and proline metabolism
Nicotinate and nicotinamide metabolism
Xylene degradation
Chlorocyclohexane and chlorobenzene degradation
Metabolism of Terpenoids and Polyketides
Fluorobenzoate degradation
Bisphenol degradation
Glutathione metabolism
Styrene degradation
Tropane, piperidine and pyridine alkaloid biosynthesis
Secondary bile acid biosynthesis
Oxidative phosphorylation
Benzoate degradation
Penicillin and cephalosporin biosynthesis
D−Glutamine and D−glutamate metabolism
Biotin metabolism
Brassinosteroid biosynthesis
Toluene degradation
Ethylbenzene degradation
DDT degradation
beta−Lactam resistance
Polycyclic aromatic hydrocarbon degradation
[B] Cytochrome P450
Nitrotoluene degradation
Metabolism of Other Amino Acid
Xenobiotics Biodegradation and Metabolism
Metabolism of xenobiotics by cytochrome P450
Drug metabolism − cytochrom P450
Drug metabolism − other enzymes
PMA_t30

Figure S20: iPath of *E. coli* K12 MG1655 metabolic KEGG map during PMA exposure at 30 min. Generated using iPath2.0 [8]. Up-regulated genes are red, down-regulated genes are blue, and increased line thickness indicates greater fold-change.

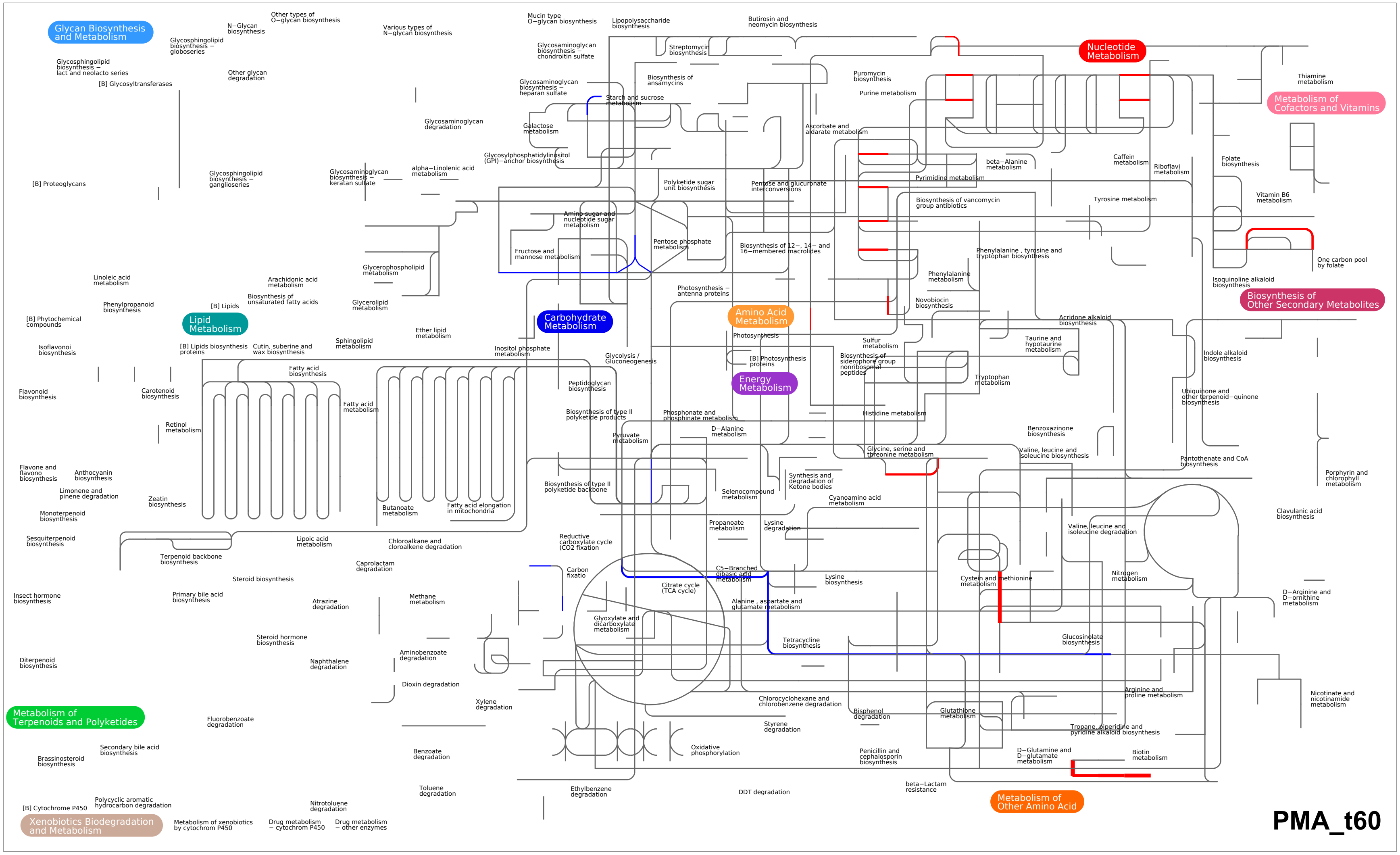
Glycan Biosynthesis and Metabolism
N−Glycan biosynthesis
Other types of O−glycan biosynthesis
Various types of N−glycan biosynthesis
Mucin type O−glycan biosynthesis
Lipopolysaccharide biosynthesis
Butirosin and neomycin biosynthesis
Glycosphingolipid biosynthesis − globoseries
Glycosphingolipid biosynthesis − lact and neolacto series
[B] Glycosyltransferases
Other glycan degradation
Glycosaminoglycan biosynthesis − chondroitin sulfate
Streptomycin biosynthesis
Nucleotide Metabolism
Glycosaminoglycan biosynthesis − heparan sulfate
Biosynthesis of ansamycins
Puromycin biosynthesis
Purine metabolism
Thiamine metabolism
Metabolism of Cofactors and Vitamins
Starch and sucrose metabolism
Glycosaminoglycan degradation
Galactose metabolism
Ascorbate and aldarate metabolism
Glycosylphosphatidylinositol (GPI)−anchor biosynthesis
beta−Alanine metabolism
Caffein metabolism
Riboflavi metabolism
Folate biosynthesis
[B] Proteoglycans
Glycosphingolipid biosynthesis − ganglioseries
Glycosaminoglycan biosynthesis − keratan sulfate
alpha−Linolenic acid metabolism
Polyketide sugar unit biosynthesis
Pentose and glucuronate interconversions
Pyrimidine metabolism
Biosynthesis of vancomycin group antibiotics
Tyrosine metabolism
Vitamin B6 metabolism
Amino sugar and nucleotide sugar metabolism
Pentose phosphate metabolism
Fructose and mannose metabolism
Biosynthesis of 12−, 14− and 16−membered macrolides
Phenylalanine , tyrosine and tryptophan biosynthesis
One carbon pool by folate
Linoleic acid metabolism
Arachidonic acid metabolism
Glycerophospholipid metabolism
Phenylalanine metabolism
Isoquinoline alkaloid biosynthesis
Photosynthesis − antenna proteins
Biosynthesis of Other Secondary Metabolites
Phenylpropanoid biosynthesis
[B] Lipids
Biosynthesis of unsaturated fatty acids
Glycerolipid metabolism
Novobiocin biosynthesis
[B] Phytochemical compounds
Lipid Metabolism
Carbohydrate Metabolism
Amino Acid Metabolism
Acridone alkaloid biosynthesis
Ether lipid metabolism
Photosynthesis
Isoflavonoid biosynthesis
[B] Lipids biosynthesis proteins
Cutin, suberine and wax biosynthesis
Sphingolipid metabolism
Inositol phosphate metabolism
Glycolysis / Gluconeogenesis
Sulfur metabolism
Taurine and hypotaurine metabolism
Indole alkaloid biosynthesis
[B] Photosynthesis proteins
Biosynthesis of siderophore group nonribosomal peptides
Fatty acid biosynthesis
Energy Metabolism
Tryptophan metabolism
Flavonoid biosynthesis
Carotenoid biosynthesis
Peptidoglycan biosynthesis
Ubiquinone and other terpenoid−quinone biosynthesis
Fatty acid metabolism
Biosynthesis of type II polyketide products
Phosphonate and phosphinate metabolism
Histidine metabolism
Retinol metabolism
D−Alanine metabolism
Benzoxazinone biosynthesis
Pyruvate metabolism
Glycine, serine and threonine metabolism
Valine, leucine and isoleucine biosynthesis
Pantothenate and CoA biosynthesis
Flavone and flavonol biosynthesis
Anthocyanin biosynthesis
Synthesis and degradation of Ketone bodies
Porphyrin and chlorophyll metabolism
Limonene and pinene degradation
Biosynthesis of type II polyketide backbone
Selenocompound metabolism
Zeatin biosynthesis
Butanoate metabolism
Fatty acid elongation in mitochondria
Cyanoamino acid metabolism
Clavulanic acid biosynthesis
Monoterpenoid biosynthesis
Propanoate metabolism
Lysine degradation
Valine, leucine and isoleucine degradation
Sesquiterpenoid biosynthesis
Lipoic acid metabolism
Chloroalkane and chloroalkene degradation
Reductive carboxylate cycle (CO2 fixation)
Terpenoid backbone biosynthesis
Caprolactam degradation
Carbon fixatio
C5−Branched dibasic acid metabolism
Lysine biosynthesis
Cystein and methionine metabolism
Nitrogen metabolism
Steroid biosynthesis
Citrate cycle (TCA cycle)
Insect hormone biosynthesis
Primary bile acid biosynthesis
Atrazine degradation
Methane metabolism
Alanine , aspartate and glutamate metabolism
D−Arginine and D−ornithine metabolism
Glyoxylate and dicarboxylate metabolism
Steroid hormone biosynthesis
Tetracycline biosynthesis
Glucosinolate biosynthesis
Diterpenoid biosynthesis
Naphthalene degradation
Aminobenzoate degradation
Dioxin degradation
Arginine and proline metabolism
Xylene degradation
Chlorocyclohexane and chlorobenzene degradation
Nicotinate and nicotinamide metabolism
Metabolism of Terpenoids and Polyketides
Fluorobenzoate degradation
Bisphenol degradation
Glutathione metabolism
Styrene degradation
Tropane, piperidine and pyridine alkaloid biosynthesis
Secondary bile acid biosynthesis
Oxidative phosphorylation
Brassinosteroid biosynthesis
Benzoate degradation
Penicillin and cephalosporin biosynthesis
D−Glutamine and D−glutamate metabolism
Biotin metabolism
Toluene degradation
Ethylbenzene degradation
DDT degradation
beta−Lactam resistance
Polycyclic aromatic hydrocarbon degradation
[B] Cytochrome P450
Nitrotoluene degradation
Metabolism of Other Amino Acid
Xenobiotics Biodegradation and Metabolism
Metabolism of xenobiotics by cytochrom P450
Drug metabolism − cytochrom P450
Drug metabolism − other enzymes
PMA_t60

Figure S21: iPath of *E. coli* K12 MG1655 metabolic KEGG map during PMA exposure at 60 min. Generated using iPath2.0 [8]. Up-regulated genes are red, down-regulated genes are blue, and increased line thickness indicates greater fold-change.

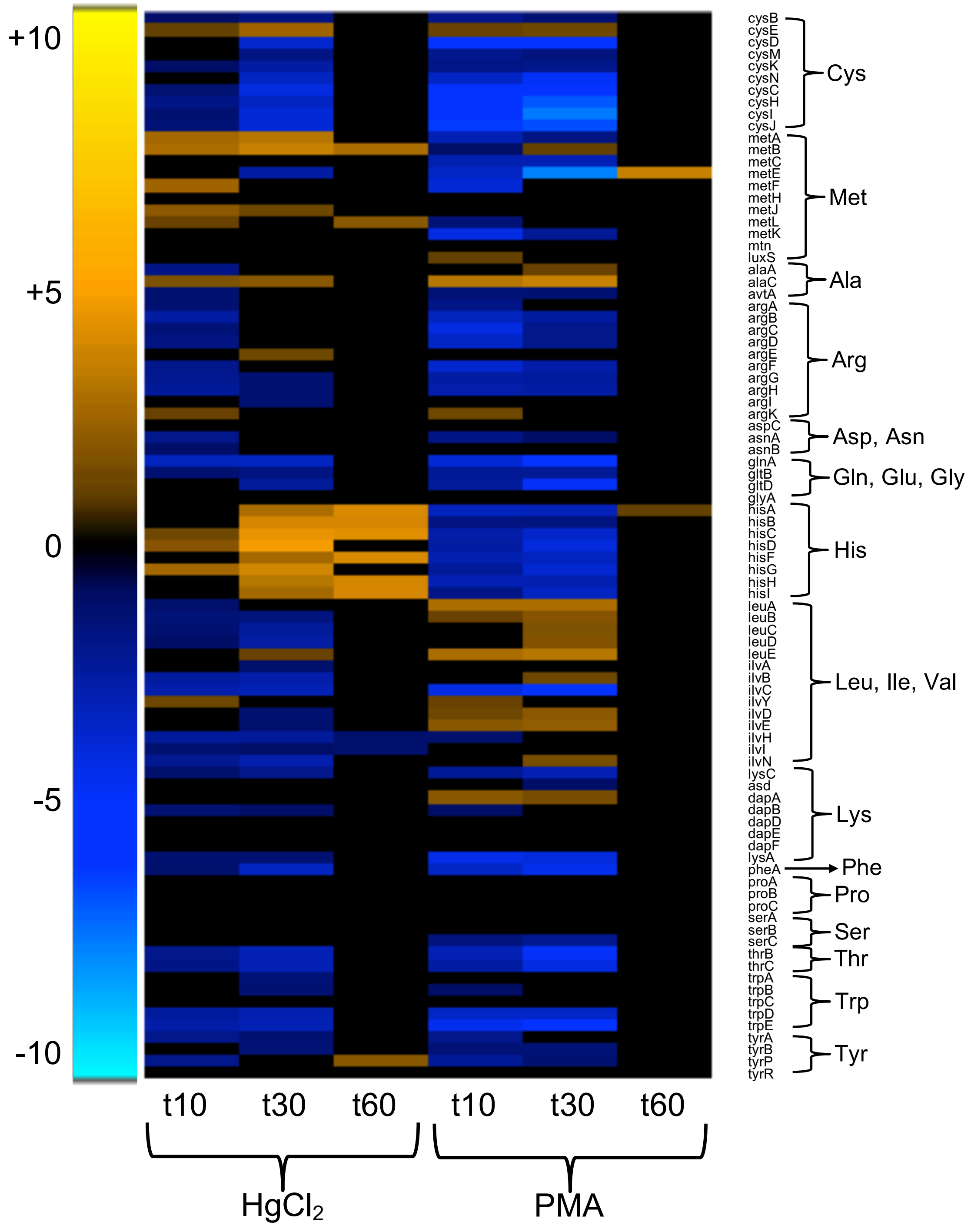

Figure S22 (Figure 8): Amino acid biosynthesis. (See Table S13 for details)

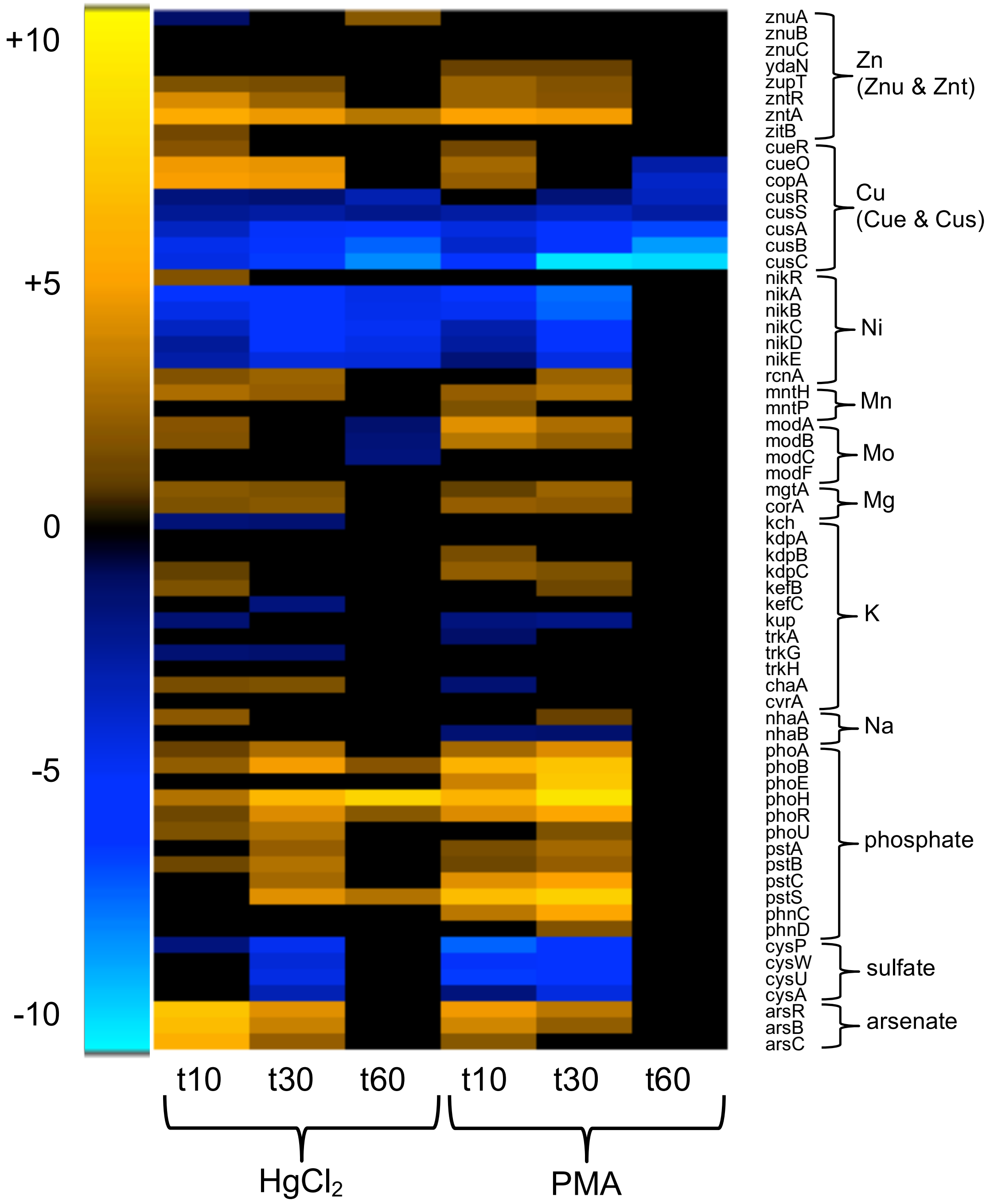

Figure S23 (Figure 9): Non-ferrous metals homeostasis. (See Table S13 for details)

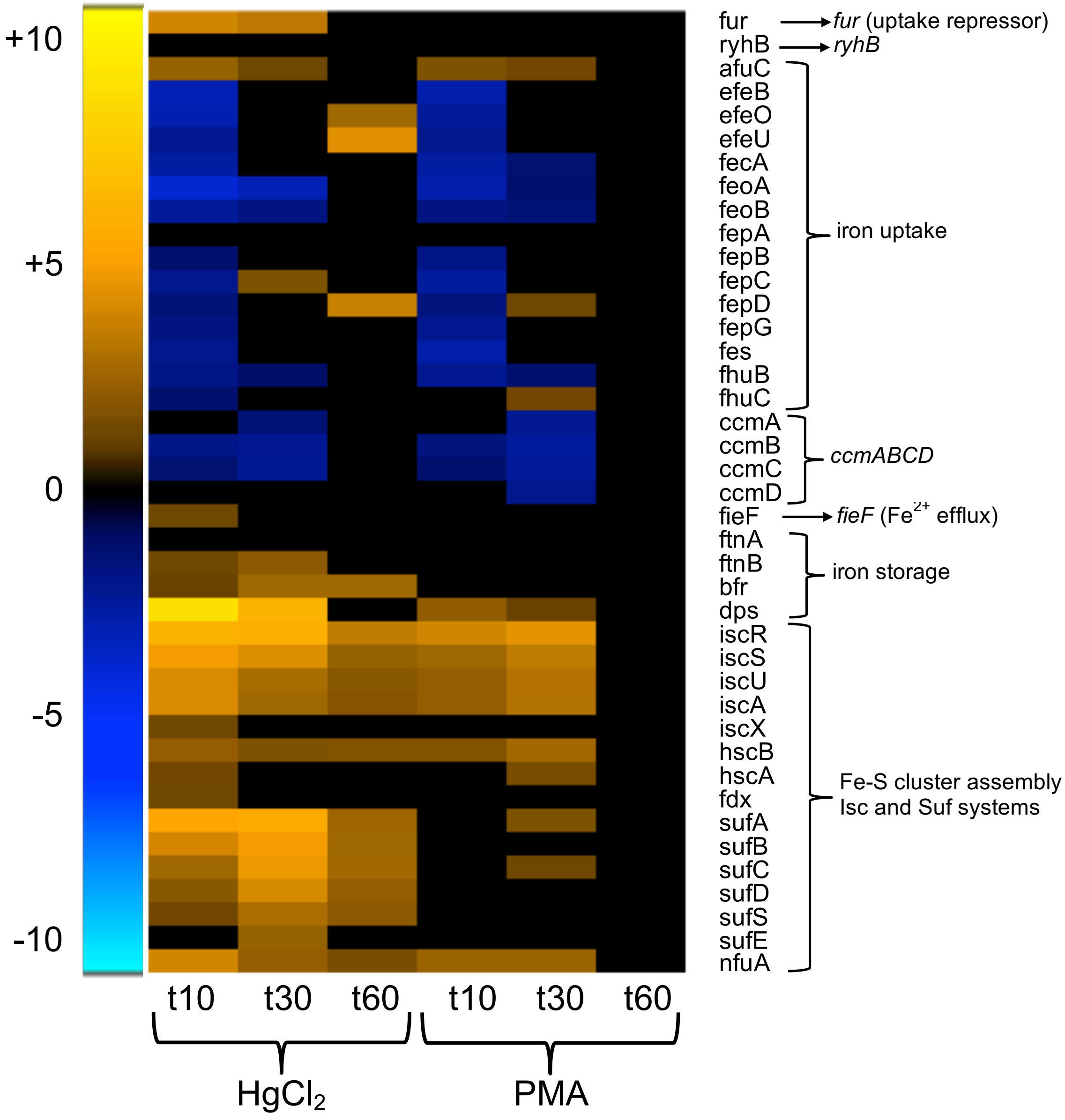

Figure S24 (Figure 10): Iron homeostasis. (See Table S13 for details)

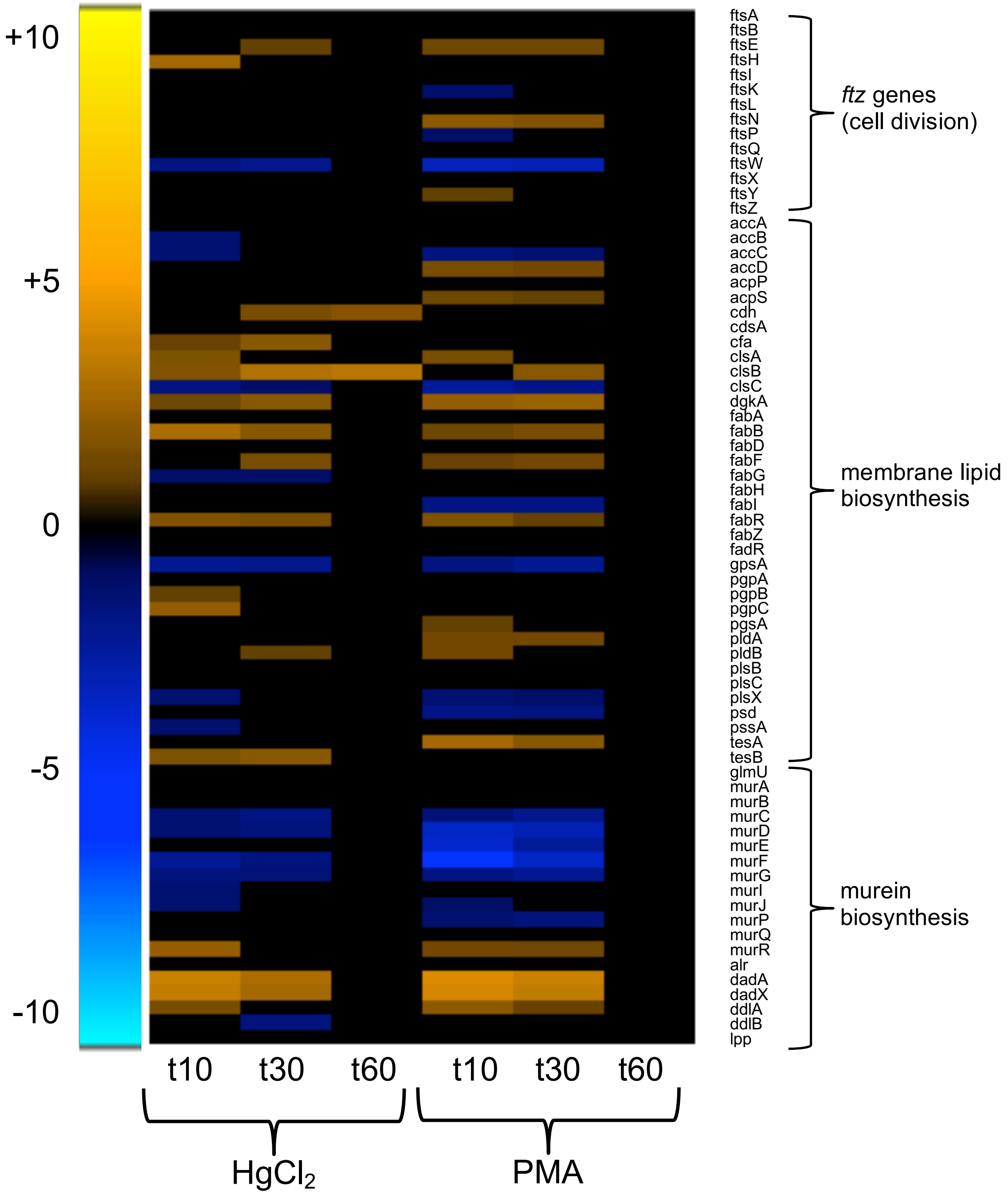

Figure S25: Cell division and cell wall biosynthesis. (See Table S13 for details)

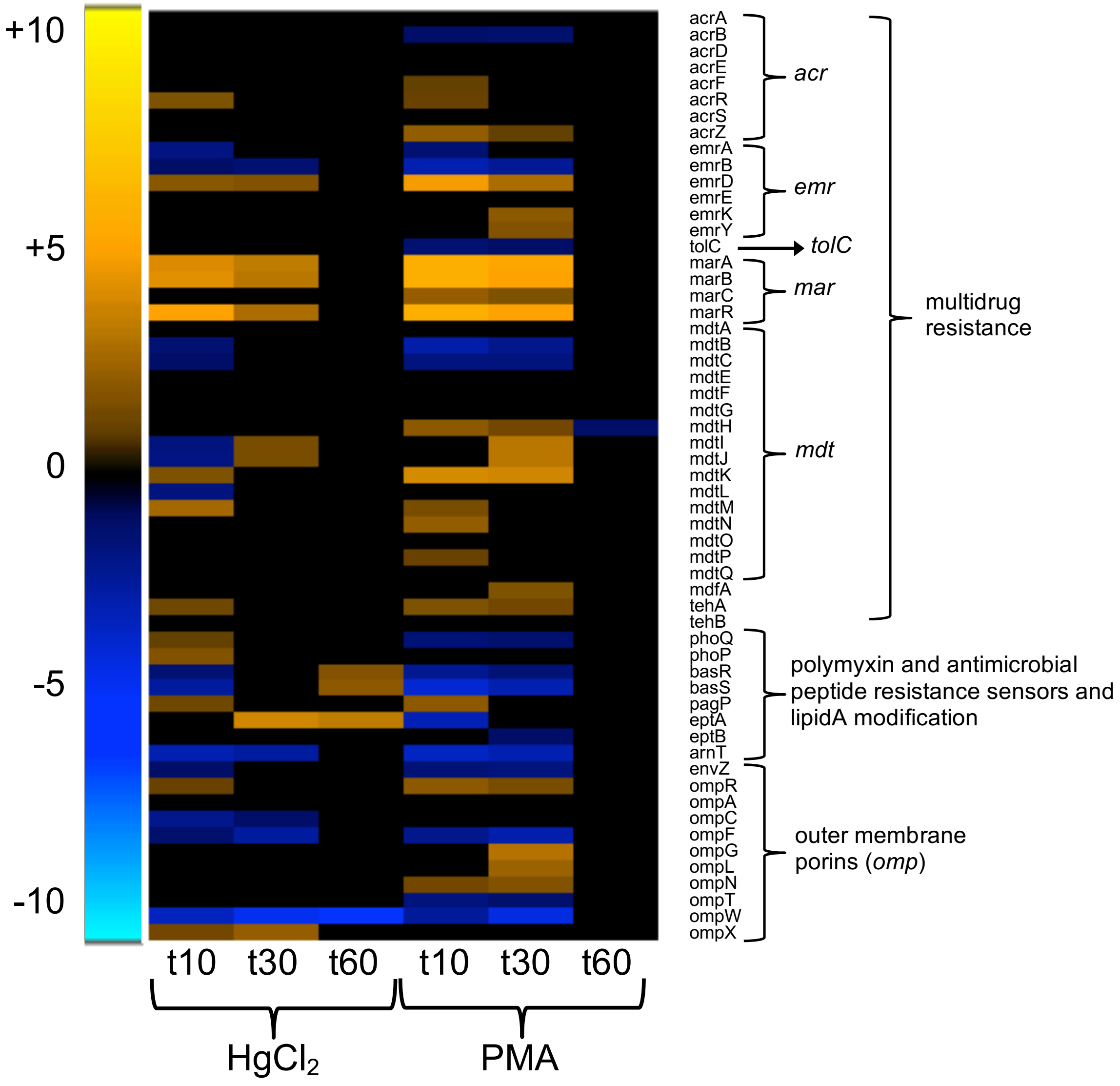

Figure S26 (Figure 11): Antibiotic resistance and outer membrane porins. (See Table S13 for details)

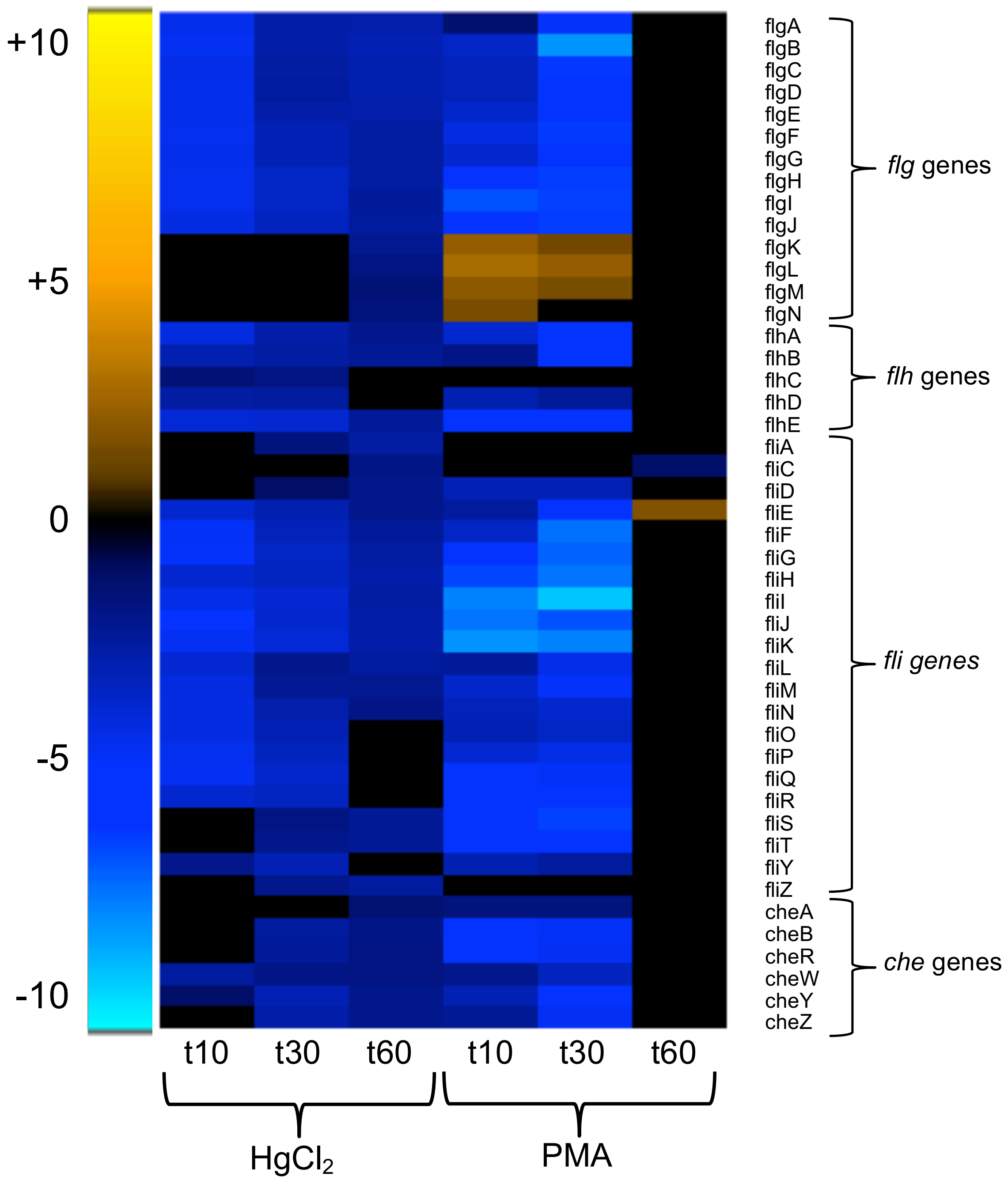

Figure S27 (Figure 12): Flagella components and chemotaxis. (See Table S13 for details)

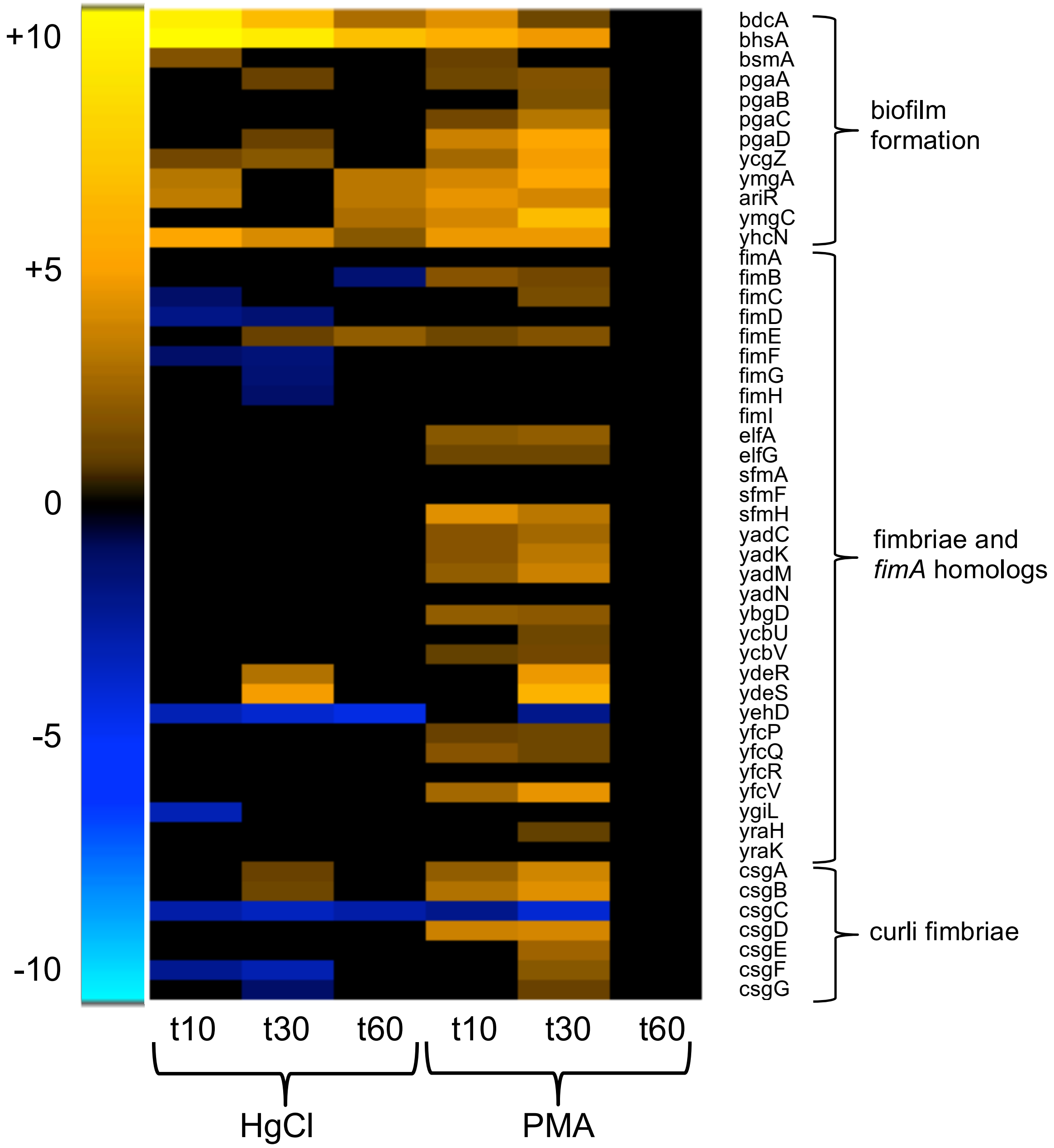

Figure S28 (Figure 13): Biofilm formation and fimbriae. (See Table S13 for details)

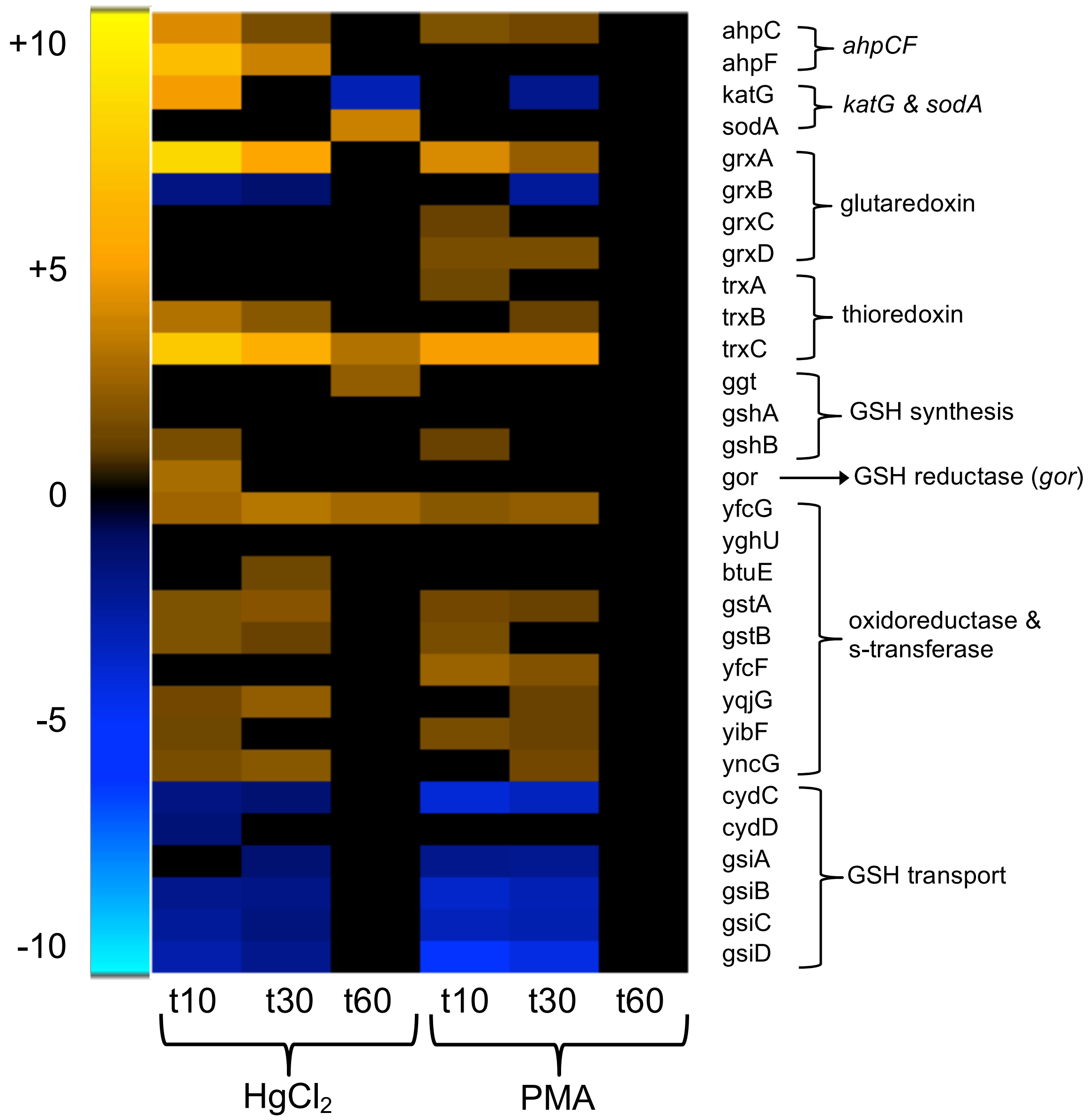

Figure S29 (Figure 14): Oxidative stress defense and thiol homeostasis. (See Table S13 for details)

SUPPORTING REFERENCES:

1. Stead MB, Agrawal A, Bowden KE, Nasir R, Mohanty BK, Meagher RB, Kushner SR. RNAsnap: a rapid, quantitative and inexpensive, method for isolating total RNA from bacteria. *Nucleic acids research.* 2012;40(20):e156.
2. Galperin MY, Makarova KS, Wolf YI, Koonin EV. Expanded microbial genome coverage and improved protein family annotation in the COG database. *Nucleic acids research.* 2015;43(Database issue):D261-269.
3. Szklarczyk D, Franceschini A, Wyder S, Forslund K, Heller D, Huerta-Cepas J, Simonovic M, Roth A, Santos A, Tsafou KP *et al*. STRING v10: protein-protein interaction networks, integrated over the tree of life. *Nucleic acids research.* 2015;43(Database issue):D447-452.
4. Salgado H, Martinez-Flores I, Lopez-Fuentes A, Garcia-Sotelo JS, Porron-Sotelo L, Solano H, Muniz-Rascado L, Collado-Vides J. Extracting regulatory networks of Escherichia coli from RegulonDB. *Methods Mol Biol.* 2012;804:179-195.
5. Lemke JJ, Sanchez-Vazquez P, Burgos HL, Hedberg G, Ross W, Gourse RL. Direct regulation of Escherichia coli ribosomal protein promoters by the transcription factors ppGpp and DksA. *Proc Natl Acad Sci U S A.* 2011;108(14):5712-5717.
6. Seo SW, Kim D, Szubin R, Palsson BO. Genome-wide Reconstruction of OxyR and SoxRS Transcriptional Regulatory Networks under Oxidative Stress in Escherichia coli K-12 MG1655. *Cell Rep.* 2015;12(8):1289-1299.

7. Keseler IM, Mackie A, Peralta-Gil M, Santos-Zavaleta A, Gama-Castro S, Bonavides-Martinez C, Fulcher C, Huerta AM, Kothari A, Krummenacker M *et al*. EcoCyc: fusing model organism databases with systems biology. *Nucleic acids research.* 2013;41(Database issue):D605-612.

8. Yamada T, Letunic I, Okuda S, Kanehisa M, Bork P. iPath2.0: interactive pathway explorer. *Nucleic acids research.* 2011;39(Web Server issue):W412-415.
